# Insights into the structure and function of Est3 from the *Hansenula polymorpha* telomerase

**DOI:** 10.1038/s41598-020-68107-x

**Published:** 2020-07-06

**Authors:** Nikita M. Shepelev, Sofia S. Mariasina, Alexey B. Mantsyzov, Alexander N. Malyavko, Sergey V. Efimov, Olga A. Petrova, Elena V. Rodina, Maria I. Zvereva, Olga A. Dontsova, Vladimir I. Polshakov

**Affiliations:** 1 M.V. Lomonosov Moscow State University, Moscow, 119991 Russia; 2 Skolkovo Institute of Science and Technology, Moscow, 121205 Russia; 3 Faculty of Biology and Biotechnology, Higher School of Economics, Moscow, 101000 Russia; 4 NMR Laboratory, Institute of Physics, Kazan Federal University, Kazan, 420008 Russia

**Keywords:** NMR spectroscopy, DNA-binding proteins, RNA-binding proteins, Transferases, Telomeres

## Abstract

Telomerase is a ribonucleoprotein enzyme, which maintains genome integrity in eukaryotes and ensures continuous cellular proliferation. Telomerase holoenzyme from the thermotolerant yeast *Hansenula polymorpha*, in addition to the catalytic subunit (TERT) and telomerase RNA (TER), contains accessory proteins Est1 and Est3, which are essential for in vivo telomerase function. Here we report the high-resolution structure of Est3 from *Hansenula polymorpha* (HpEst3) in solution, as well as the characterization of its functional relationships with other components of telomerase. The overall structure of HpEst3 is similar to that of Est3 from *Saccharomyces cerevisiae* and human TPP1. We have shown that telomerase activity in *H. polymorpha* relies on both Est3 and Est1 proteins in a functionally symmetrical manner. The absence of either Est3 or Est1 prevents formation of a stable ribonucleoprotein complex, weakens binding of a second protein to TER, and decreases the amount of cellular TERT, presumably due to the destabilization of telomerase RNP. NMR probing has shown no direct in vitro interactions of free Est3 either with the N-terminal domain of TERT or with DNA or RNA fragments mimicking the probable telomerase environment. Our findings corroborate the idea that telomerase possesses the evolutionarily variable functionality within the conservative structural context.

## Introduction

Telomerase is an enzyme essential for the synthesis and replication of telomeres—nucleoprotein structures capping the ends of eukaryotic chromosomes. Telomerase’s core enzyme is a reverse transcriptase containing a protein catalytic subunit (TERT) and telomerase RNA (TER)^1–3^. Using a portion of TER as a template, TERT elongates the 3′-ends of telomeric single-stranded DNA, synthesizing a string of telomeric repeats^4^. The action of telomerase compensates for the loss of telomeric DNA through successive cell divisions due to the end-replication problem^5–8^. In human, most differentiated somatic cells lack telomerase activity. In highly proliferative cells, e.g. unicellular eukaryotes and embryonic cells of mammals, telomerase expression and activity is tightly controlled, although the exact mechanisms of this regulation are poorly understood^9–11^ (see also^12^ for a recent review). The malfunction of these mechanisms and up-regulation of telomerase is the leading cause of cell immortalization in most types of cancer^13–16^. However, the understanding of telomerase function and regulation remains a challenge due to limited amount of structural information on the organization of the telomerase complex and the interactions of its components.

In vivo the function of telomerase, in addition to the core enzyme, requires accessory protein subunits, which vary considerably from one species to another (reviewed in Ref.^17^), and their precise role in the regulation of telomerase is still not completely understood. In budding yeast *Saccharomyces cerevisiae* such accessory telomerase subunits are Est1, Est3, a heptameric ring of Sm proteins, yKu heterodimer, and a recently discovered set of the Pop proteins (Pop1/Pop6/Pop7)^18–26^. Sm_7_ binds near the 3′-end of the TER and is required for its stability and maturation^21^. yKu is implicated in the nuclear import of the telomerase RNA, and plays role in the association of telomerase with telomeres^18–20^. Pop1/Pop6/Pop7 stimulate association of TERT and Est1 with telomerase RNA in vivo^22,23^. They are dispensable for TERT activity in vitro, and their precise role in the regulation of telomerase is still not completely understood. Deletion of either *EST1* or *EST3* leads to gradual telomere loss and loss of viability identical to the Est2 and TLC1 (homologs of TERT and TER in *S. cerevisiae*) null mutants^25^, yet telomerase isolated from *∆est1* or *∆est3* strains was still active in vitro^27^. Est1 contains an RNA binding domain and interacts directly with a specific stem within telomerase RNA^28,29^. Est1 also directly binds the telomeric ssDNA-binding protein Cdc13, and this interaction is crucial for telomerase recruitment at telomeres^30–33^. Apart from its recruitment function, Est1 is also required for loading Est3 into the telomerase complex^34–36^. Est3 is a small protein unable to bind telomerase RNA directly and has to rely on protein–protein interactions to become a part of the telomerase holoenzyme. In addition to Est1, Est3 was shown to interact in *S. cerevisiae* with Est2, specifically with its N-terminal domain (TEN)^35,37–39^. It was found that Est3 associates with the preassembled Est1-TLC1-Est2 subcomplex with maximum binding observed only late in the cell cycle (late G2/M), suggesting that Est3 loading is a highly regulated step during telomerase assembly^35^. However, the mechanism of telomerase activation by Est3 association still remains unclear.

In other budding yeast species (*Saccharomyces castellii* and *Candida albicans*) the requirement for Est3 protein for telomerase activity in vitro appears to be more pronounced than in *S. cerevisiae*^38,40^. Interestingly, in the case of *C. albicans*, the effect of *EST3* deletion depends on the primer used in the telomerase assay^40^. Whether these observations point to mechanistic distinctions in Est3 functions in different species is yet to be determined. However, deletion of *EST1* in *C. albicans* leads to an almost identical defect^41^. Remarkably, in the *est3*-null strain, CaEst1 loses the ability to bind telomerase RNA (the same is true for the *est1*-null strain and CaEst3), further stressing differences in telomerases from diverse species^40^.

The structure of *S. cerevisiae*’s Est3 protein (lacking 12 N-terminal amino acids) was obtained using a strategy that combines minimal NMR experimental data with Rosetta de novo structure prediction algorithm^42^. This study revealed that ScEst3 is an OB-fold, as was predicted earlier^43^; however, the experimentally obtained structure differs significantly from the predicted model. The unbiased mutagenesis of every surface residue defined a surface which is important for the association of ScEst3 with the telomerase complex^42^. Although the human telomerase complex does not contain an obvious homologue of Est3, one human telomeric protein—TPP1—has an OB-fold domain^44^ structurally very similar to ScEst3. hTPP1 was shown to be important for telomerase recruitment to telomeres via interaction with the TEN domain of human TERT, and the residues responsible for the interaction cluster in a single surface (dubbed the TEL patch)^45,46^. Most strikingly, the identified surface of ScEst3 almost coincides with the TEL patch on hTPP1, suggesting the functional importance of the Est3/TPP1—TEN interaction for telomerase action^42^.

Previously we published the ^1^H, ^13^C and ^15^N resonance assignments of HpEst3^47^. Here we report the high-resolution solution structure of HpEst3 along with genetic and biochemical characterization of this protein. We believe that this information is essential for a deeper understanding of the mechanism of telomerase function and regulation.

## Results

### *Hansenula polymorpha* Est3 is essential for telomerase activity

**Figure 1:**
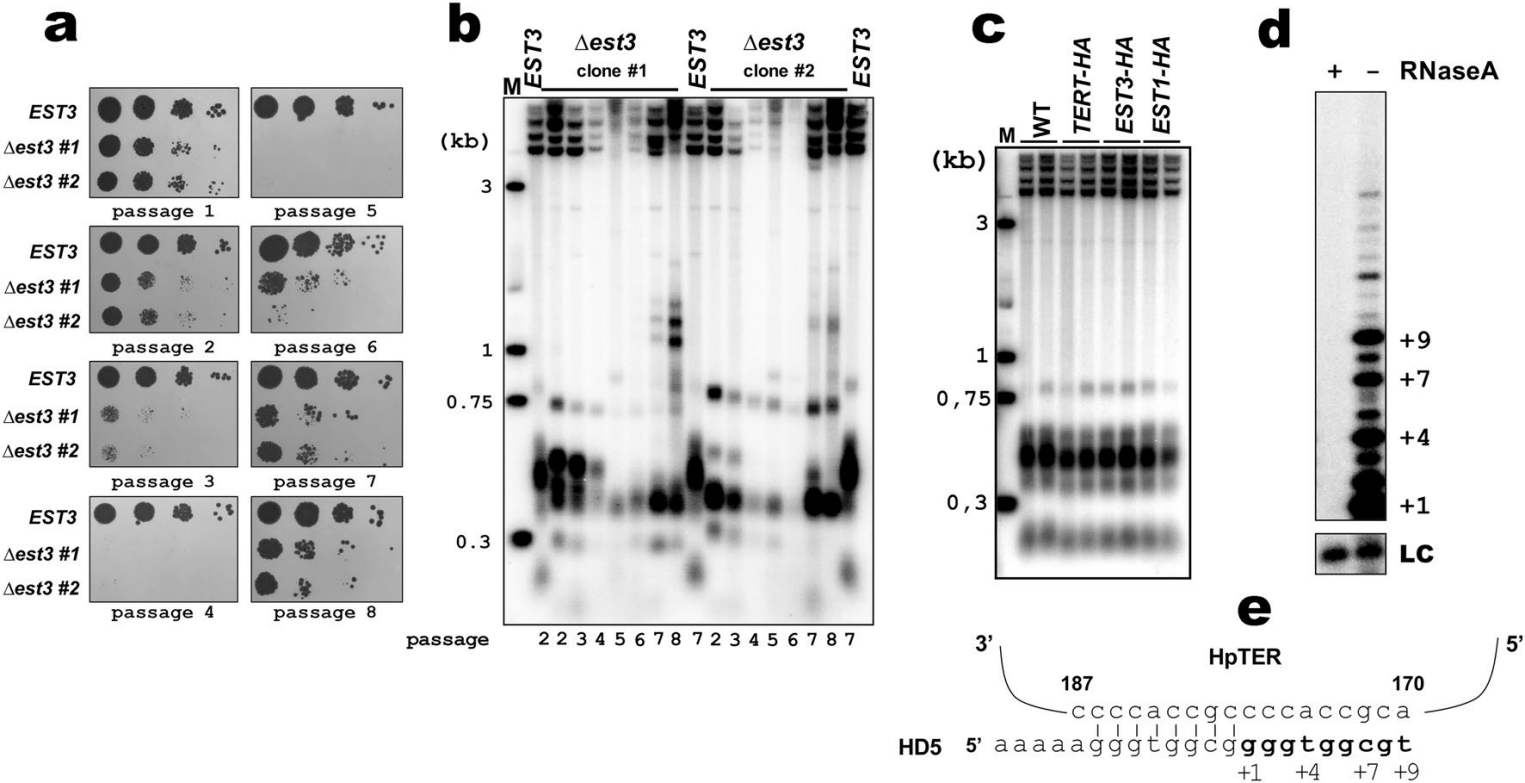
(**a**) Spot assay. *EST3* (wild type) and *∆est3* (two clones isolated after transformation) strains were passaged in liquid SC + LEU (*EST3*) or SC-LEU (*∆est3*) medium. Cell culture aliquots (and three serial tenfold dilutions) from the indicated passage were spotted onto a YPD plate, grown for 2 days and photographed. (**b**) Southern blot analysis of terminal restriction fragments from the indicated strains. Genomic DNA was isolated from cells after each passage (passage number is shown under each lane). (**c**) Same as (**b**), however DNA used for this analysis was isolated from yeast cultures after 10 restreaks on agar plates (~ 200 generations). (**d**) Telomerase activity assay of the *TERT-HA EST3* IP sample [isolated from 400 ml of YPD culture (OD_600_ = 1)] with HD5 primer, radiolabeled dGTP and unlabeled dTTP, dCTP, dATP. Positions of + 1, + 4, + 7 and + 9 elongation products are indicated; faint bands longer than + 9 most likely correspond to products resulting from translocation of the + 8 and the second round of the template copying (type II translocation). “LC” loading control (a [γ-^32^P]-labeled 13-mer oligomer). (**e**) Schematic of HD5 primer alignment along the template region of the HpTER (nucleotides 170–187). Nucleotides added by telomerase in the in vitro assay are in bold. Original (full lane view, no contrast adjustment) Southern blots and gels from (**b**, **c** and **d**) are shown in Supplementary Fig. S14.

To confirm that the identified HpEst3 homologue is required for telomerase action we constructed a *H. polymorpha* strain (*∆est3*) with Est3 open reading frame substituted for *HpLEU2* marker. *∆est3* strain was propagated in liquid YPD medium for several days. As it was described for knock-outs of other *H. polymorpha* telomerase components (HpTER, HpTERT and HpEst1)^48,49^, *∆est3* cells rapidly lost telomeric DNA and exhibited greatly reduced viability at the earliest passages (Fig. 1a, b). A population of “survivors” subsequently emerged, which maintain their telomeres presumably via recombination.

To isolate telomerase and to investigate the possibility that Est3 is necessary for telomerase activity in vitro, we also constructed a strain expressing HpTERT tagged with a hemagglutinin epitope (*TERT-HA*). The tagged TERT behaves as the native protein, as judged by the telomere length analysis (Fig. 1c). Extract prepared from the *TERT-HA EST3* strain was incubated with anti-HA agarose, and telomerase activity precipitated on the beads was assessed by its ability to elongate 13-mer oligonucleotide HD5 in the presence of radiolabelled dGTP. We detected several elongation products (up to + 9) expected from the HpTER template sequence (Fig. 1d, e).

**Figure 2:**
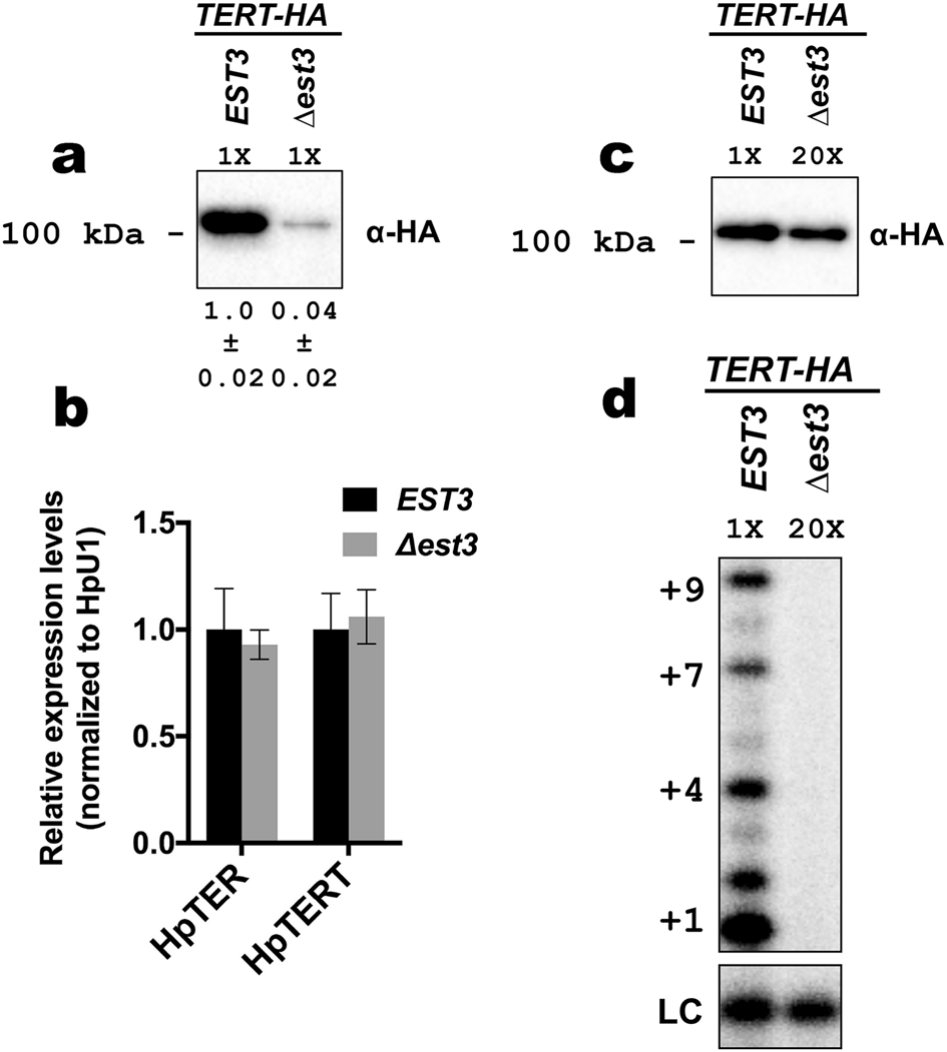
(**a**) Western blot analysis (with anti-HA antibodies) of TERT-HA protein levels in anti-HA precipitates prepared from the indicated strains. 1/2 portions of the IP sample obtained from 400 ml YPD cultures (OD_600_ ~ 1) were used for analysis. Numbers below are quantifications of band intensities (mean ± SD); data collected from experiments with three independently grown cultures of each strain. (**b**) Expression levels of telomerase RNA (HpTER) and HpTERT mRNA in *TERT-HA EST3* and *TERT-HA ∆est3* strains as determined by quantitative RT-PCR. qPCR signals from test RNAs were normalized to HpU1 snRNA as described in METHODs. Data obtained using four RNA preparations are plotted in the diagram (mean ± SD). (**c**) anti-HA Western blot analysis shows comparable amount of TERT-HA protein in 1/10 portion of the IP sample obtained from 400 ml YPD culture (OD_600_ ~ 1) of *TERT-HA EST3* and in 1/2 portion of the IP sample obtained from 1,600 ml YPD culture (OD_600_ ~ 1) of *TERT-HA ∆est3*. The equivalent amounts of IP samples were analyzed by primer extension assay (in **d**). (**d**) Telomerase activity assay of *TERT-HA EST3* and *TERT-HA ∆est3* IP samples (containing similar amount of TERT) with HD5 primer. Telomerase elongation products (up to + 9 nucleotides) are visible in *TERT-HA EST3* sample, but not in *TERT-HA ∆est3*. “LC” loading control (a [γ-^32^P]-labeled 13-mer oligomer). Data in (**c** and **d**) are representative of three independent experiments. Original (full lane view, no contrast adjustment) blots and gels from (**a**, **c** and **d**) are shown in Supplementary Figs. S14 and S15.

Surprisingly, the amount of TERT-HA precipitated from the *TERT-HA ∆est3* strain was reduced ~ 25-fold compared to the parental *TERT-HA EST3* strain (in which the *EST3* gene is intact), suggesting that the Est3 protein is required for the normal accumulation of TERT protein in *H. polymorpha* cells (Fig. 2a). We did not detect any reduction in TERT mRNA abundance upon deletion of the *EST3* gene; therefore, Est3 influences either translation process or TERT protein stability (Fig. 2b). Of note, HpTER RNA levels are identical in *EST3* and *∆est3* strains; therefore, not all telomerase subunits are downregulated after Est3 loss (Fig. 2b). Taking into account this effect we compared telomerase preparations from *EST3* and *∆est3* containing a similar amount of TERT-HA protein in primer elongation assay. Despite the presence of TERT at comparable levels in both preparations (Fig. 2c), we did not detect any nucleotide addition by the telomerase isolated from the *TERT-HA ∆est3* strain (Fig. 2d). Unfortunately, we could not reliably determine the HpTER content of the two samples, because the amount of HpTER co-purified with TERT-HA from the *∆est3* strain was very small (close to background levels), leading to large experimental error. Collectively, results from this section show that Est3 is an essential protein for telomerase action in *H. polymorpha* and is required for the normal accumulation of TERT protein.

### Est3 structure determination

**Figure 3:**
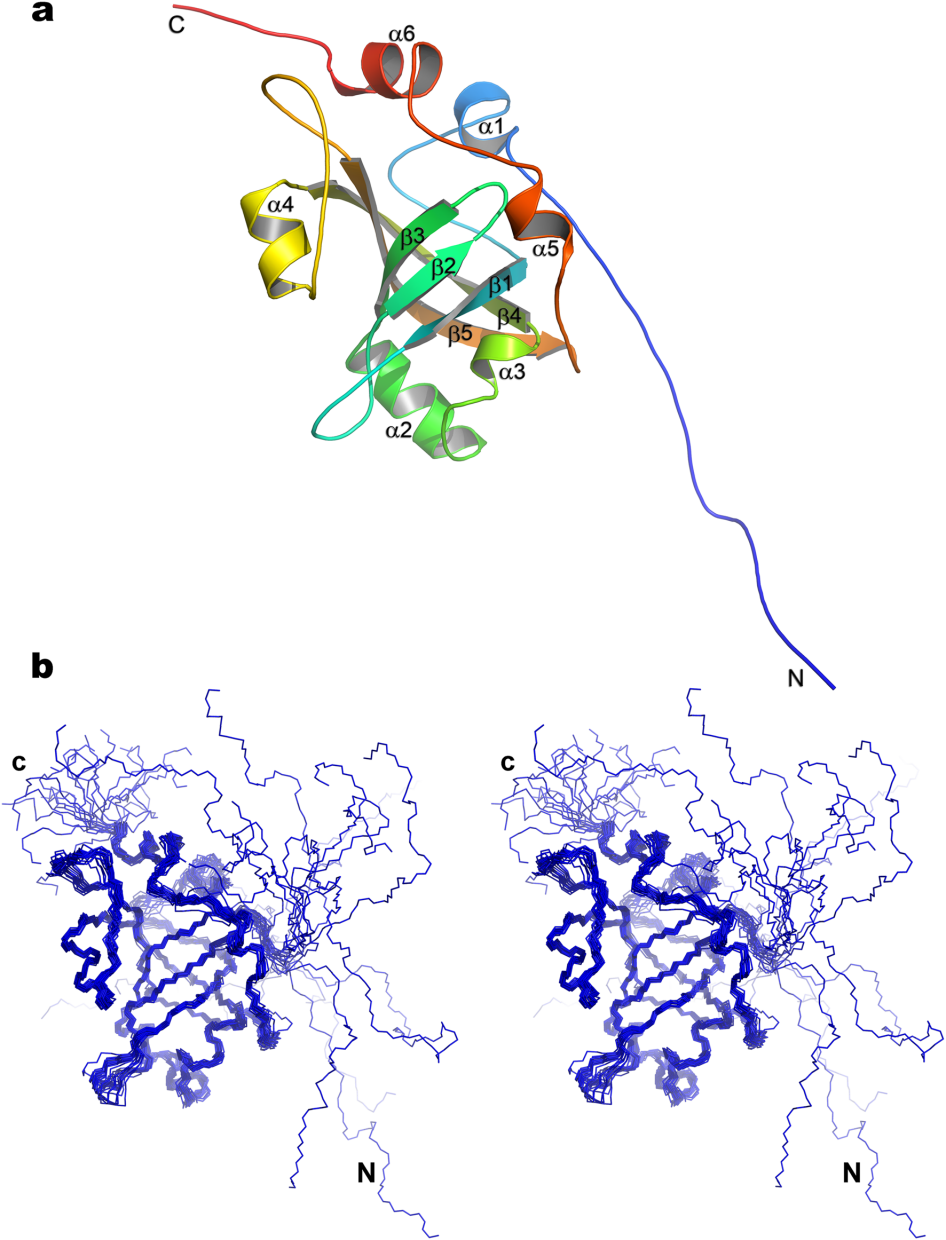
The solution structure of the HpEst3. (**a**) The topology of the secondary structure elements of the HpEst3 protein. (**b**) The stereo view of the ensemble of the final 20 calculated structures. Images were made using PyMOL v. 2.3 (Schrodinger, LLC).

The NMR signal assignments of the HpEst3 protein have been reported earlier^47^. A family of 20 NMR structures was determined using 2,490 experimental restraints measured at 298 and 303 K (see Table 1 for details). This work made use of standard double and triple resonance NMR methods applied to ^15^N and ^15^N/^13^C labeled samples of HpEst3. For most of the protein residues, the number of NOEs per residue is between 20 and 40 (Supplementary Fig. S1). This number is less for the N and C-terminal residues and residues from several protein loops. The protein consists of the structured β-barrel core (residues 22–172) and disordered N and C-terminal tails (residues 1–22 and 173–178) (see Fig. 3b). Five β-strands surrounded by six α-helices and several loops form the protein core (Fig. 3a). The protein core is well structured: root-mean-square deviation (RMSD) of the coordinates of heavy backbone atoms of the residues 22–172 in the family of 20 NMR conformers is 0.87 ± 0.14 Å (Table 1). In the Ramachandran plot analysis (Supplementary Fig. S2), 87% of the residues in the whole NMR family were found in the most favored regions and none in the disallowed regions.

**Table 1:** Statistics for the ensemble of the calculated 20 structures of the HpEst3.

A. Restraints used in the structure calculation			
Total NOEs	2,262	Total dihedral angles	228
Long range (|i − j|> 4)	443	Phi (ϕ)	116
Medium (1 < |i − j|≤ 4)	210	Psi (ψ)	112
Sequential (|i − j|= 1)	568		
Intraresidue	1,041		

B. Restraint violations and structural statistics (for 20 structures)		
Average RMSD	< S > ^a^	S_rep_
**From experimental restraints**		
Distance (Å)	0.042 ± 0.002	0.041
Dihedral (°)	0.269 ± 0.053	0. 253
**From idealized covalent geometry**		
Bonds (Å)	0.0021 ± 0.0001	0.0019
Angles (°)	0.3540 ± 0.0102	0.3500
Impropers (°)	0.2380 ± 0.0145	0.2420
**Ramachandran plot statistics**		
% of residues in most favorable region of Ramachandran plot	81.3	87.0
% of residues in disallowed region of Ramachandran plot	0.0	0.0

C. Superimposition on the representative structure (Å)	
Backbone (C, Cα, N) RMSD over the structured protein core (residues 22–172)	0.87 ± 0.14

No NOE or dihedral angle violations are above 0.5 Å and 5° respectively.

^a^< S > is the ensemble of 20 final structures; S_rep_ is the representative structure, selected from the final family on the criteria of having the lowest sum of pairwise RMSD for the remaining structures in the family.

The structure of HpEst3 adopts a classic OB-fold topology^50,51^ similar to that of Est3 from *Saccharomyces cerevisiae*^42^ or human TPP1^44,52^ (see “Discussion”). The family of HpEst3 structures and experimental restraints used in solution structure calculations have been deposited in the Protein Data Bank under accession number 6Q44.

### Protein backbone dynamics

**Figure 4:**
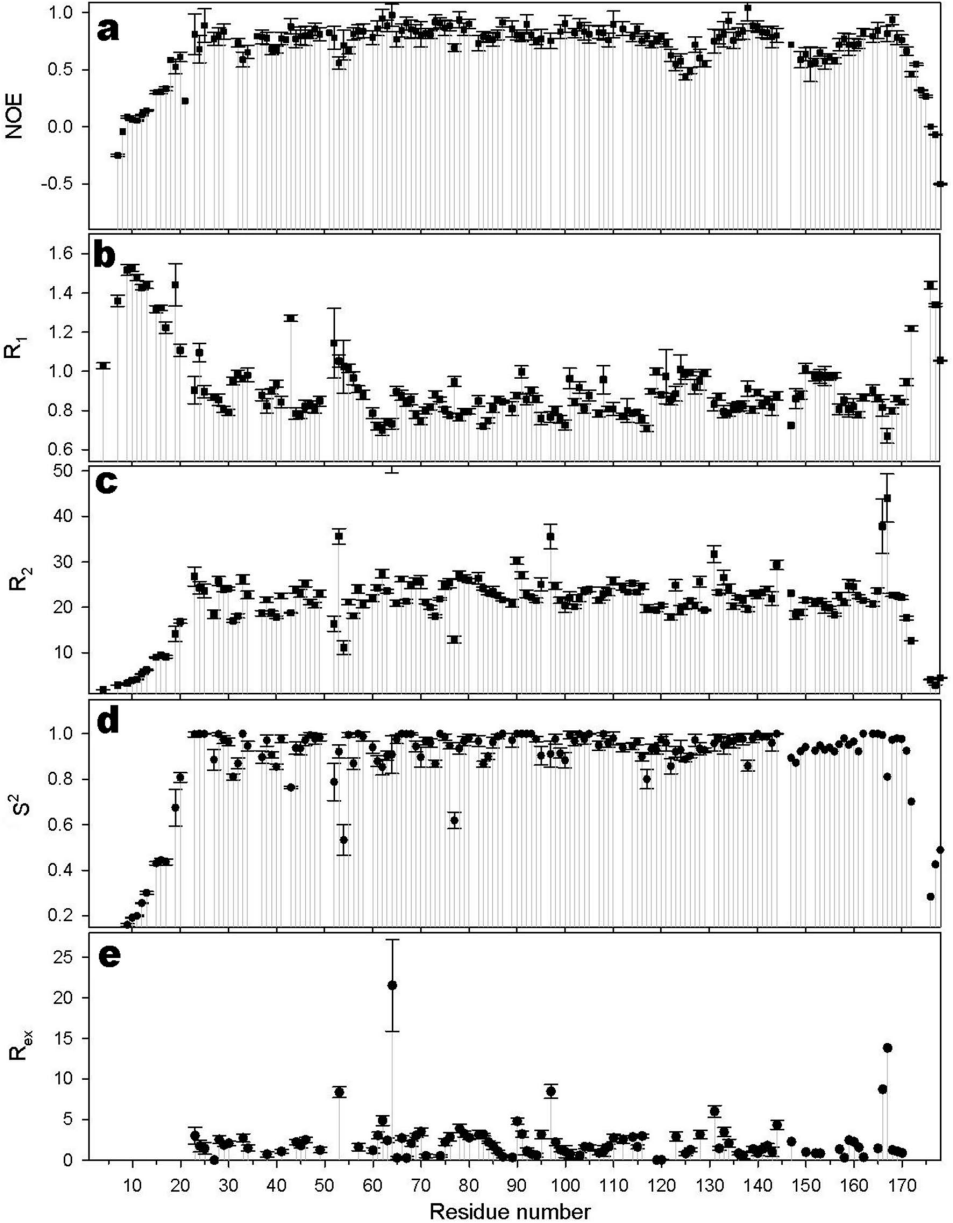
The relaxation parameters of the amide ^15^N nuclei of each residue of the HpEst3, measured at 16.3 T (700 MHz proton resonance frequency) and 298 K. (**a**) The heteronuclear ^15^ N,^1^H-steady-state NOE values. (**b**) The longitudinal relaxation rate *R*_1_ (s^−1^). (**c**) The transverse relaxation rate *R*_2_ (s^−1^). (**d**) The order parameter *S*^2^, determined by model-free analysis. (**e**) Chemical exchange *R*_*ex*_ contributions to the transverse relaxation rates (s^−1^).

**Figure 5:**
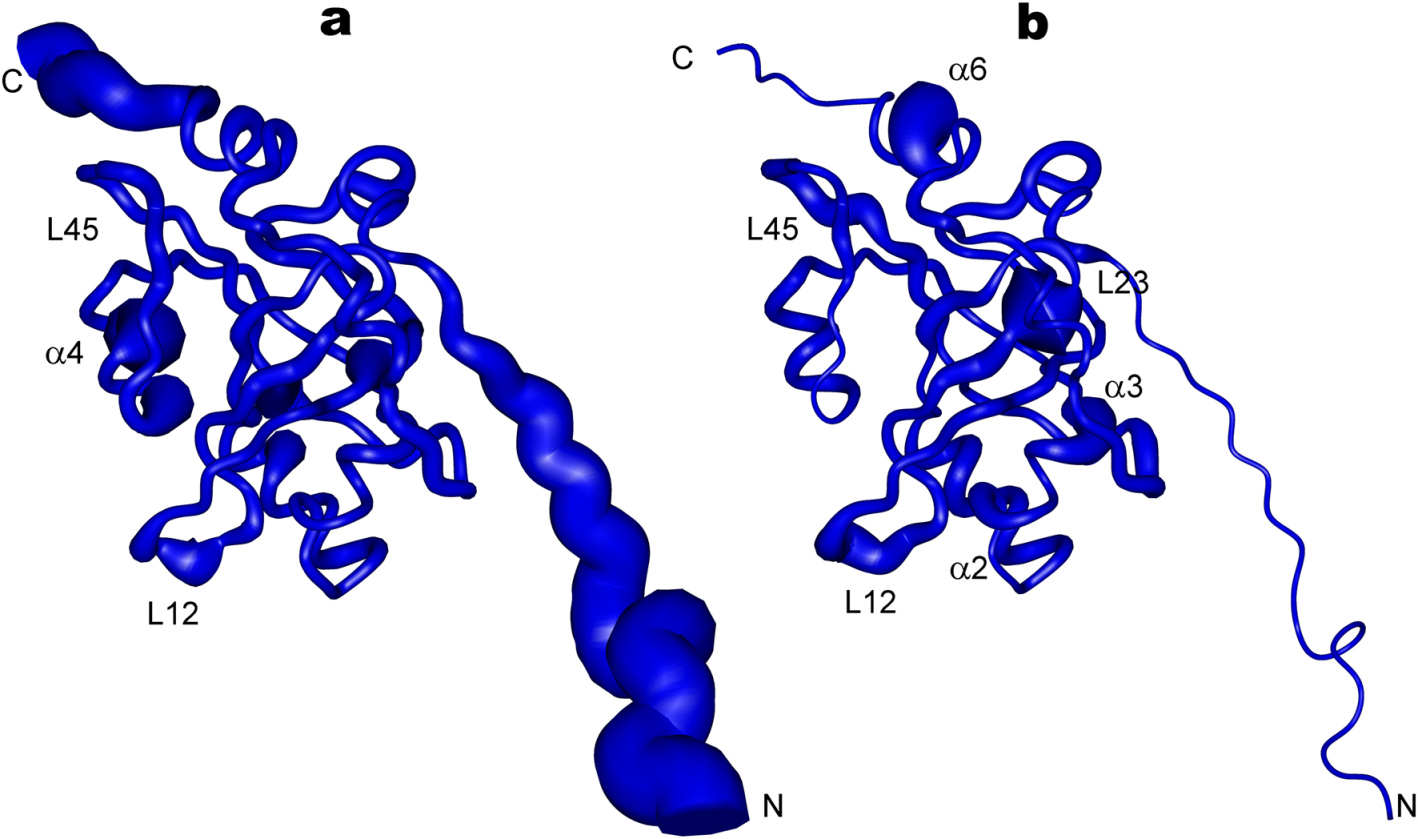
A cylindrical ribbon representation of the backbone of the C domain of the HpEst3. The variable radius/thickness of the cylinder is proportional to the dynamic properties. (**a**) Motions in the ps to ns time scale. Thickness of the cylinder is proportional to the value of (1 − S^2^); the minimal thickness corresponds to the value S^2^ = 1, the maximum to S^2^ = 0.15. (**b**) Motions in the ms time scale. Thickness of the cylinder is proportional to the value of R_ex_; the minimal thickness corresponds to the value R_ex_ = 0, the maximum to R_ex_ = 20. Figure was made using the Insight II v. 2000 software (Molecular Simulations Inc.).

Dynamic properties of the HpEst3 backbone in ps-ns and ms time scales were studied using the ^15^N relaxation parameters for the amide ^15^N nuclei. Longitudinal (R_1_) and transverse (R_2_) relaxation rates measured at 298 K and heteronuclear ^15^N-^1^H Overhauser effects are shown on Fig. 4a–c. Model-free analysis of the experimental data allowed us to obtain values of the order parameter S^2^ (Fig. 4d), which reflects the amplitude of ps-ns amide bond vector dynamics and chemical exchange contribution to the transverse relaxation rate R_ex_ (Fig. 4e), manifesting protein motions occurring in the ms time scale. The value of the correlation time of protein tumbling *τ*_*m*_ calculated from the experimental R_2_/R_1_ ratios is 13.6 ± 2.0 ns. Applying models of anisotropic motions only slightly improves the data fit. Thus, for the axial anisotropy model, the ratio of the principal axes of the anisotropy tensor (D^║^/D^┴^) is less than 1.2. Therefore, model-free analysis of relaxation data has been carried out in the assumption of isotropic motion. As expected, residues from the unstructured N- and C-terminal tails undergo fast internal motions resulting in small values of S^2^. There are several loops which have increased internal mobility in the ps-ns time scale (Fig. 5a). Among them are the loop L12 (residues 50–59), the loop L45, which separates helix α4 and strand β5 (residues 118–131), and the residues 144–161 between strand β5 and helix α6, including helix α5 in the middle (see Fig. 4d). However, the most interesting feature of Est3 is the protein dynamics in ms time scale, associated with an exchange between two or more conformations (Fig. 5b). Almost all protein residues of the structured protein core are involved in such a conformational exchange (Fig. 4e). The values of R_ex_ for most of the amide ^15^N nuclei are between 2 and 10 Hz and maximum values observed for the residues 53, 64, 97, 131, 166, 167. These correspond to the fragments of helices α2, α3 and α6 and loops L12, L23 and L45 (Fig. 5b). Conformational broadening of the Est3 resonances is clearly observed in NMR spectra recorded at lower temperature (Supplementary Fig. S3). A decrease in temperature of only 10–15 °C results in a significant broadening of all the Est3 resonances except the unstructured tails. Many signals are disappearing in the spectra measured at 288 K. This indicates that 288 K just slightly exceeds the coalescence temperature of the chemical exchange for the most such signals.

### Probing the interaction of Est3 with components of telomerase and telomeres

The possible interaction of HpEst3 with partners in the formation of the telomerase complex was probed by heteronuclear NMR spectroscopy. The interactions of Est3 with TEN, as well as with the single-stranded DNA fragments corresponding to telomeric repeats, RNA constructs, corresponding to TER fragments, and RNA–DNA heteroduplexes were investigated (see Supplementary Table S1). Interactions were monitored by the changes of ^1^H and ^15^N chemical shifts of HpEst3 upon the increase of the concentration of each tested binding partner (see Supplementary Figs. S4–S11). To study the binding of HpEst3-TEN, two complementary experiments were carried out, one of which tested a change in the chemical shifts of HpEst3, and the other—TEN. In none of the cases specific binding of HpEst3 with nucleic acids or TEN has been observed. When HpEst3 interaction with ssDNA fragment that corresponds to four telomeric repeats (G4) was monitored, protein binding to DNA was observed, but it was not specific. With an addition of long single-stranded DNA to protein, a significant broadening of the HpEst3 signals was observed, indicating the formation of high molecular weight complexes (Supplementary Fig. S6). However, no noticeable changes in chemical shifts characteristic of specific binding occurs.

We also tested the ability of Est3 to bind DNA in electrophoretic mobility shift assay, using fluorescently labeled DNA oligonucleotide containing four telomeric repeats (fG4) as a probe. We did not observe any bands corresponding to Est3-fG4 complex even at relatively large concentrations of protein and DNA (10 μM and 1 μM, respectively) (Supplementary Fig. S12a). Small portion of fluorescent signal remained trapped in the gel wells, which probably correspond to non-specific aggregation observed in the NMR experiment.

### Association of Est3 protein with telomerase RNA is Est1-dependent, but TERT-independent

**Figure 6:**
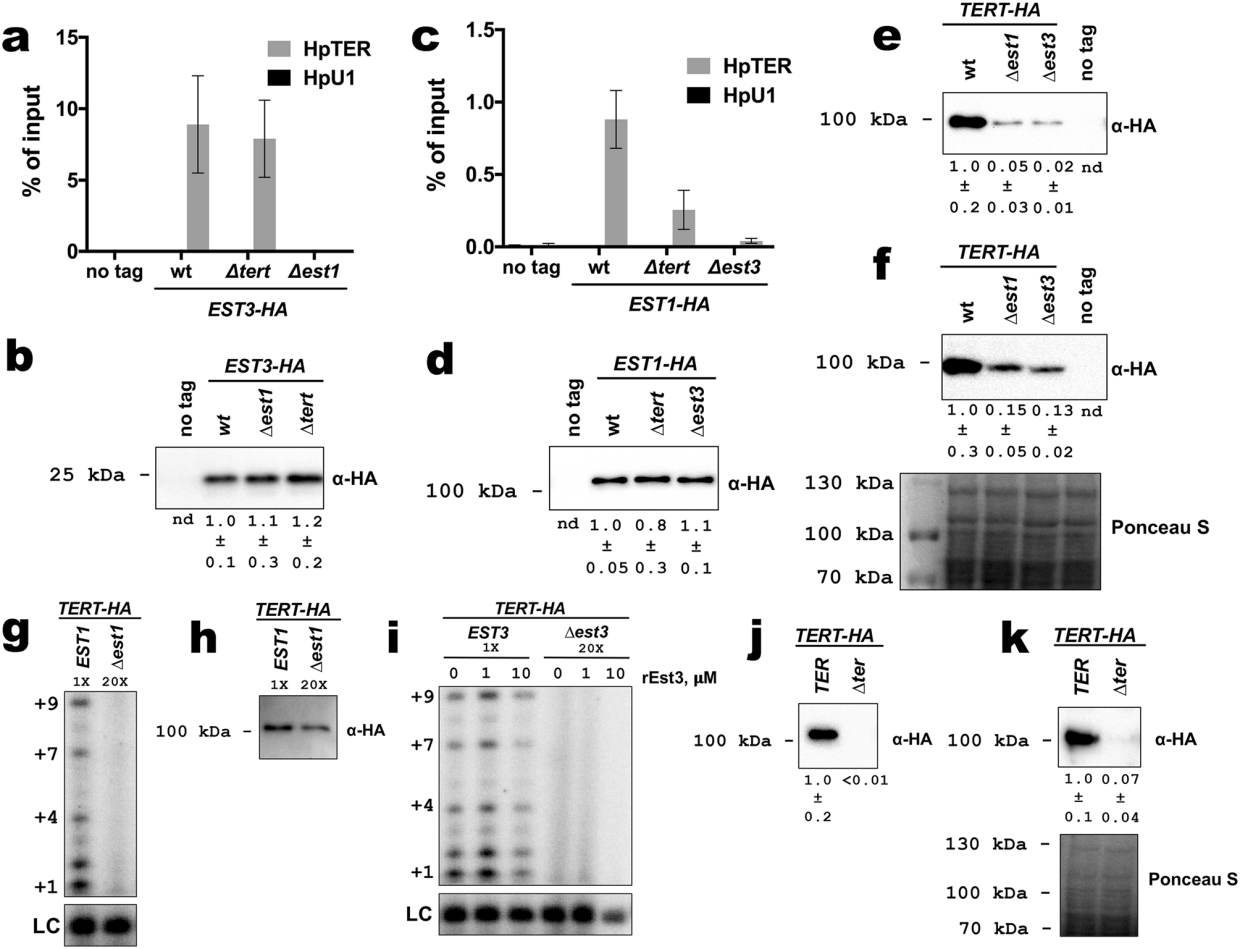
(**a**) Analysis of HpTER association with Est3-HA. Quantitative RT-PCR analysis of the HpTER co-precipitated on anti-HA agarose after incubation with extracts from the indicated strains. Small nuclear RNA HpU1 was also analyzed as a negative control. Data represented as percentage of input RNA (mean ± SD). (**b**) Western blot analysis (with anti-HA antibodies) of Est3-HA protein levels in anti-HA precipitates prepared from the equal amounts of cultures of the indicated strains. Numbers below are quantifications of band intensities (mean ± SD) calculated using values from three biological replicates. “nd”—not determined. Extracts prepared from three independently grown cultures of each strain were utilized for the experiments in (**a**) and (**b**). (**c**) and (**d**) same as (**a**) and (**b**), respectively, only with Est1-HA protein. (**e**) same as (**b**) but with TERT-HA protein. 1/2 portions of the IP sample obtained from 400 ml YPD cultures (OD_600_ ~ 1) were used for analysis. (**f**) Anti-HA Western blot analysis of TERT-HA protein levels in extracts prepared from the indicated strains (upper panel). Ponceau S-stained membrane served as a loading control (lower panel). Equal amounts of input samples were used for analysis. Numbers below the blot are quantifications of band intensities (mean ± SD) calculated using values from three biological replicates. “nd”—not determined. (**g**) Telomerase activity assay of *TERT-HA EST1* and *TERT-HA ∆est1* IP samples with HD5 primer. “LC” loading control. 1/10 portion of the IP sample obtained from 400 ml YPD culture (OD_600_ ~ 1) of *TERT-HA EST1* and in 1/2 portion of the IP sample obtained from 1,600 ml YPD culture (OD_600_ ~ 1) of *TERT-HA ∆est1* were used for the experiment. (**h**) Western blot analysis of samples from (**g**). (**i**) same (**g**) but with *TERT-HA EST3* and *TERT-HA ∆est3* IP samples. Telomerase on beads was pre-incubated with recombinant Est3 (rEst3) (1 or 10 μM) where indicated. (**j**) same as (**e**) but with *TERT-HA ∆ter* strain. (**k**) same as (**f**) but with *TERT-HA ∆ter* strain. Original (full lane view, no contrast adjustment) blots and gels from (**b**, **d**–**k**) are shown in Supplementary Fig. S16.

Our inability to detect interaction between HpEst3 and HpTEN in vitro prompted us to check which components are necessary for recruitment of Est3 into the telomerase complex in *H. polymorpha*. To monitor Est3-HpTER association we constructed a strain (*EST3-HA*) expressing tagged Est3 from its native genomic locus. The *EST3-HA* allele is functional: no major telomere shortening was observed in the strain (Fig. 1c), and HpTER robustly co-elutes with Est3 following immunoprecipitation (IP) on anti-HA agarose (Fig. 6a). Notably, the amount of HpTER coprecipitated with Est3-HA is not influenced by the absence of the *TERT* gene (Fig. 6a). In sharp contrast, deletion of the *EST1* gene completely abolishes the HpTER signal in the Est3-HA IP sample (Fig. 6a). The levels of isolated Est3-HA are identical for all three strains (*EST3-HA*, *EST3-HA* ∆*tert,* and *EST3-HA* ∆*est1*) (Fig. 6b). Thus, in *H. polymorpha*, recruitment of Est3 into the telomerase complex relies on the presence of Est1 protein, rather than its direct interaction with the TEN domain of TERT as it was observed in other species.

Similarly, we investigated the possibility that Est3 is required for Est1-HpTER interaction, using an *EST1-HA* strain [again, functionality of the tagged protein was confirmed by the telomere length analysis (Fig. 1c)]. Indeed, we found that Est1-HpTER association is effectively abolished in the absence of Est3 (Fig. 6c, d). Deletion of *TERT* also reduces the amount of HpTER co-immunoprecipitated with Est1-HA, however this effect is less pronounced than in the case of *EST3* deletion (Fig. 6c). Therefore, Est1 and Est3 proteins rely on each other to bind HpTER.

The ~ tenfold difference in co-IP HpTER between the Est1-HA and Est3-HA WT samples (Fig. 6a, c) apparently contradicts this latter statement, suggesting that a large portion of cellular Est3 may associate with Est1-free (and, potentially, TERT-free) HpTER. However, we should note that the two experiments shown in Fig. 6a, c were performed using different batches of the affinity resin, which may translate to different IP efficiencies between experiments. Also, the observed effect may result from variations in epitope accessibility. Indeed, when we compared HpTER co-IP efficiencies between Est1-HA and Est3-HA WT strains processed in parallel, we found reduced Est3-HA/Est1-HA ratio (~ threefold, Supplementary Fig. S13a). Moreover, Est3-HA sample contained more telomerase activity, compared to Est1-HA, indicating that telomerase complex is more efficiently immunoprecipitated via Est3-HA (Supplementary Fig. S13b, c).

Given this interdependence of Est1 and Est3 for interaction with telomerase RNA, we tested whether the deletion of *EST1* would have any effect on the TERT protein levels, as we observed for the *est3*-null strain (Fig. 2a). Remarkably, the amount of TERT-HA is greatly reduced in the *TERT-HA ∆est1* strain and indistinguishable from the *TERT-HA ∆est3* strain [both in whole-cell extracts and in the eluates after immunoprecipitation on anti-HA agarose (Fig. 6e, f)]. Moreover, telomerase from the *TERT-HA ∆est1* strain was deficient in primer elongation in vitro (Fig. 6g, h), further emphasizing the functional link between Est1 and Est3 in *H. polymorpha*. Consistent with the absence of Est1 in telomerase preparation from the *TERT-HA ∆est3* strain, addition of recombinant Est3 did not restore in vitro defect of the *TERT-HA ∆est3* telomerase (Fig. 6i). Although, since we could not reliably determine the level of telomerase RNA in the *TERT-HA ∆est3* sample, this result may be also explained by the absence of HpTER. Finally, we discovered that deletion of HpTER leads to the same (if not stronger) defect in the TERT-HA protein accumulation (Fig. 6j, k), suggesting that TERT stabilization by Est1 and Est3 may be mediated through HpTER.

## Discussion

As expected, we found that Est3 protein is an essential subunit of *H. polymorpha* telomerase complex, required for telomeric DNA addition in vivo. We also showed that telomerase from the *∆est3* strain is defective in the primer elongation in vitro. The requirement of Est3 for in vitro telomerase activity has also been observed in other yeast systems^38–40^, although the degree of this requirement varies; and the nature of the Est3’s effect on nucleotide addition by telomerase still remains elusive.

**Figure 7:**
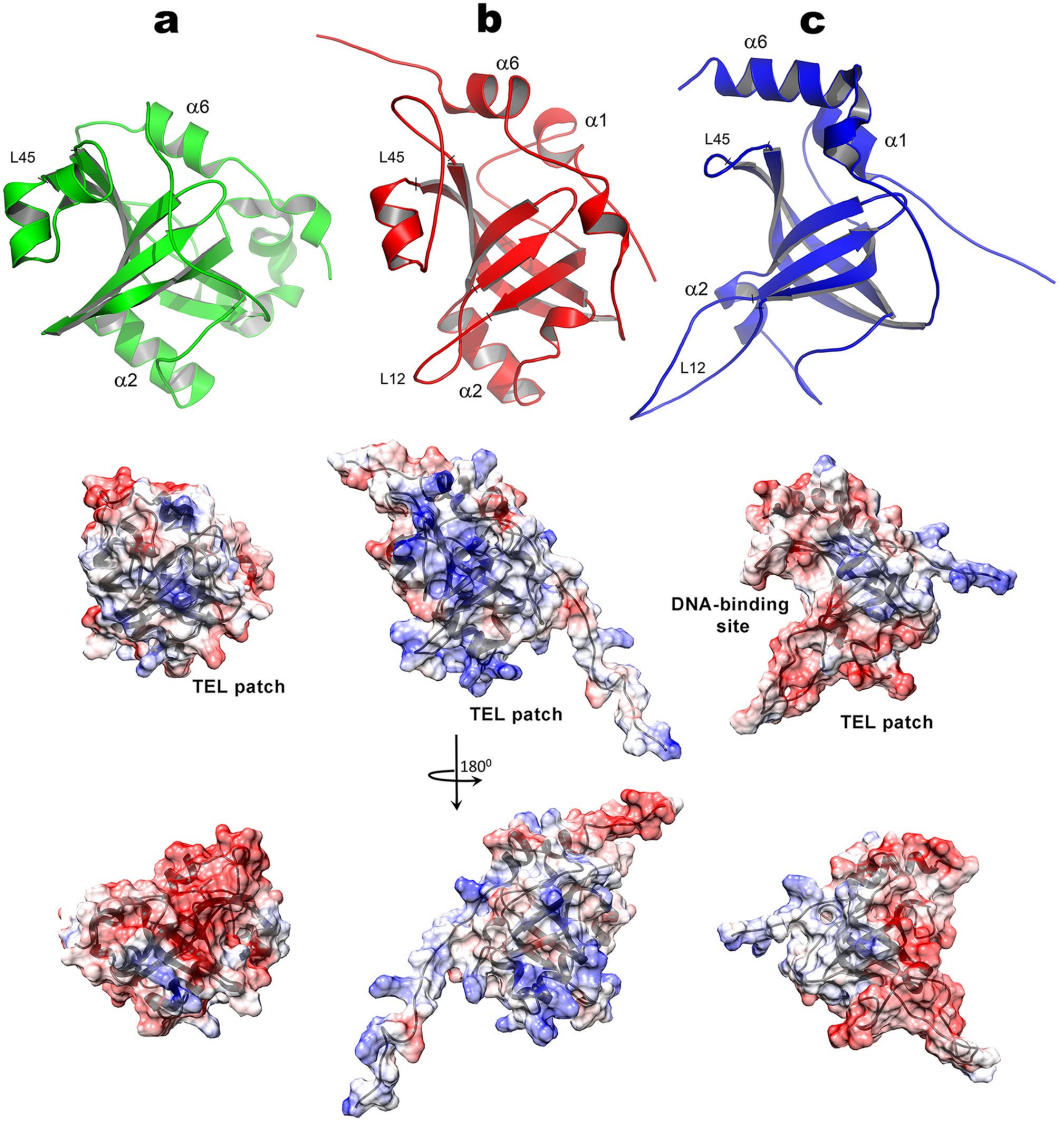
Structures (top) and Coulomb charge distribution over the molecular surfaces (bottom) of Est3 from (**a**) *Saccharomyces cerevisiae* (PDB ID 2M9V^42^), (**b**) *Hansenula polymorpha* (PDB ID 6Q44, this work), and (**c**) human TPP1 (PDB ID 2I46^44^). Images were made using PyMOL v. 2.3 (Schrodinger, LLC).

**Figure 8:**
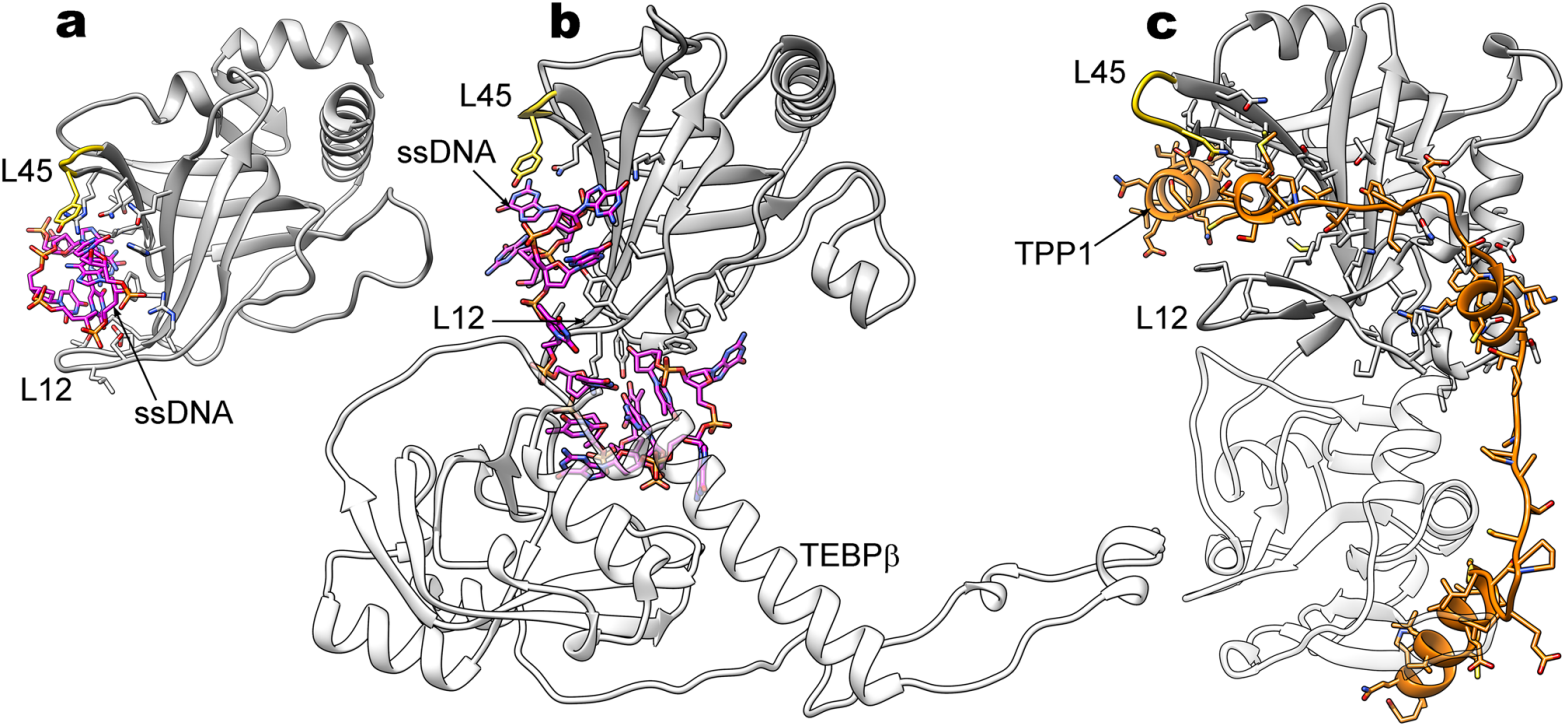
Binding interfaces of the telomeric OB fold proteins: (**a**) *S. pombe* POT1 with single-stranded telomeric DNA fragment (magenta); (**b**) N-terminal OB fold domain of *S.nova* TEBPα complexed with TEBPβ and a fragment of ssDNA (magenta); (**c**) human POT1 complexed with a peptide fragment of TPP1 (orange). The loop 45 is shown in gold. Side chains of the ligand-interacting residues of OB fold proteins are shown in sticks. Figure was made using UCSF Chimera v. 1.12 (https://www.cgl.ucsf.edu/chimera).

The determined high-resolution structure of HpEst3 showed high level of similarity with previously obtained structures of hTPP1^44^ and ScEst3^42^ (Fig. 7). They all have an OB-fold typical for oligonucleotide/oligosaccharide binding proteins^42,53^. The OB fold domains are widespread in proteins. Due to their high structural plasticity, OB fold modules may be adapted to various functionalities. The most extensively characterized is the ssDNA-binding ability of OB-fold proteins; among other known functionalities are their interactions with RNA and proteins, including other OB fold units. OB fold domains were found in a number of telomere-binding proteins from various species, where they demonstrate all these modes of interaction. The structural core of OB-fold proteins is composed of conserved beta-strand elements β1–β5 forming the two anti-parallel beta-sheets, connecting loop elements L12, L23, and L45 of variable length, and optional α-helices, the most conserved of which is a C-terminal helix (C-helix). Analysis of the architecture of telomeric OB fold-containing proteins^53^ suggests that the length and position of connecting elements and alpha-helices may reflect the interaction preferences of a particular OB fold module. Figure 8 shows the examples of the ssDNA- and protein-binding telomeric proteins^54–56^ with the focus on their interaction interfaces and the position of the connecting element L45. Typical for these proteins is a "canonical" binding surface capable of interacting with ssDNA or extended peptide fragments, defined by the loops L45 and L12 from above and below. In addition, OB modules obviously can interact with protein molecules, including other proteins or other OB modules of the same protein. Unlike the classical oligonucleotide-binding interface, this interaction surface can vary in different OB fold proteins. One of such surfaces is located below the β-barrel and is mediated with the L34 loop which in different OB fold proteins may include different secondary structure elements. It appears that, in some cases, binding of the two protein molecules or subunits is necessary for the tight interaction between OB fold domain and the oligonucleotide partner, and, vice versa, binding of DNA is necessary to form the binding interface for the protein partner. Such cooperativity can be seen in the structure of the SnTEBPα/β-DNA complex from *Sterkiella nova* (Fig. 8b) where the protein-DNA interaction site is formed in the α/β subunit interface. OB fold modules within the human heterodimer hPOT1/hTPP1 are structurally similar to the SnTEBPα/β complex. Of this pair, only hPOT1 is capable of binding ssDNA directly, but formation of a complex hPOT1/hTPP1 tightens this interaction tenfold^44,57^, which may indicate the analogous structural interdependence. In the budding yeasts, neither structural homologs of POT1 or TEBPα nor interaction between Est3 and nucleic acid partners was found. However, Est3 in *S. cerevisiae* interacts directly with Est1 and with TERT, both of which bind telomerase RNA. These interactions may also be cooperative due to the plasticity of OB fold units.

In spite of the overall structural similarity, there are important differences between HpEst3, ScEst3, and hTPP1. First of all, unlike hTPP1 and many other OB fold proteins which have a short L45 loop that does not obstruct the classical ligand-binding interface, the L45 loops in both yeast proteins (HpEst3 and ScEst3) are significantly larger (Fig. 7). This prevents the interaction with potential ligands at the canonical interface without a prior change in the loop conformation. Such conformational transitions are often observed when OB-fold proteins interact with DNA or RNA^58^. Dynamic properties of HpEst3 determined by ^15^N relaxation measurements indicate the occurrence of conformational rearrangements of the protein backbone around the canonical OB-fold ligand-binding interface (Fig. 5b). Therefore, we cannot exclude the possibility of exposing the ligand-binding interface in HpEst3 due to the conformational rearrangements of the protein molecule.

Another significant difference is seen at the conserved region of Est3 surface, the TEL patch, which in hTPP1 and ScEst3 is involved in the recruitment of telomerase subunits to the telomere through the direct interaction with the N-terminal domain of TERT^42,45,46^. The TEL regions of hTPP1 and ScEst3 surfaces are negatively charged, while the corresponding part of HpEst3 is positively charged (Fig. 7). Additionally, the second region at the N-terminus of human TPP1 (so called NOB region, residues 91–95 of hTPP1) was shown to mediate both telomerase recruitment to telomeres and repeat addition processivity^59^. This effect was sequence-specific: replacing the NOB region of human TPP1 with NOB of its close homolog, mouse TPP1, reduced processivity of human telomerase. In Est3 proteins, the corresponding region shows some limited sequence conservation within yeast, with the exception of HpEst3 and some other species, but it has no similarity with animal TPP1 proteins. These considerations may reflect differences in intermolecular interactions in which these proteins are involved.

NMR experiments showed that free HpEst3 protein does not interact specifically with oligonucleotides modeling possible DNA and/or RNA environment of Est3 in telomerase complex. It is likely that the additional binding partners are required to trigger structural changes capable to open the ligand-binding interface.

In *S. cerevisiae*, telomerase stimulation by Est3 was linked to its ability to bind the TEN-domain of the catalytic subunit^39^. Direct interaction between isolated recombinant Est3 and the TEN-domain was also reported for proteins from yeasts *Candida parapsilosis* and *Lodderomyces elongisporus*^60^. Having well-behaved recombinant HpEst3 and HpTEN, we tested the possibility of the direct interaction between these proteins by NMR. However, chemical shift changes were not observed neither in the case of titration of ^15^N-labelled Est3 by TEN, nor ^15^N-labelled TEN by Est3. These results suggest that either HpEst3 lacks the ability to bind HpTEN or such an interaction becomes possible only within the assembled *H. polymorpha* telomerase complex. The apparent absence of in vitro interaction between Est3 and TEN has also been reported by Tucey and Lundblad^35^, studying the proteins from *S. castellii* species. Tucey and Lundblad contested the idea that the Est3 binds to an isolated TEN even in *S. cerevisiae*. They showed that a stable complex between ScEst3 and ScTERT does not form unless ScTERT is pre-bound to telomerase RNA^35^.

However, in *S. cerevisiae*, Est3 does not appear to contact telomerase RNA directly—interaction with TERT/TEN has been implicated in recruiting ScEst3 to the telomerase complex in vivo^24,35,37–39^. This assumption has not been tested in any other yeast species. HpEst3, similar to ScEst3, was found to be unable to bind nucleic acids. Therefore, its association with HpTER is most likely to be mediated by protein–protein interactions. We analyzed the amount of TER co-precipitated with Est3-HA from *TERT* and *∆tert* backgrounds; however, we did not find any significant reduction of the Est3-bound TER upon TERT removal. This result is consistent with the lack of Est3—TEN binding in vitro, and suggests that TERT/TEN may not serve as a factor in recruiting Est3 to TER in *H. polymorpha*.

Association of Est3 with TER in *S. cerevisiae* has been shown to be stimulated by the Est1 protein^34–36^. Similar Est1-dependency of the Est3-TER complex formation is described for *C. albicans*^40^. Interestingly, CaEst1 is unable to form a stable complex with CaTER in the absence of CaEst3 in vivo^40^. In *S. cerevisiae*, telomerase RNA levels immunoprecipitated with Est1 are also diminished in *est3*-null mutants^24,34,61^, although to a lesser extent. We found that this interdependence of Est3 and Est1 for productive TER binding can be observed in *H. polymorpha* cells as well (Fig. 6). Thus, Est1 and Est3 are linked (at least functionally), and this connection is conserved among budding yeast species, perhaps, even more conserved than Est3-TEN interaction. Interestingly, yeasts *C. parapsilosis* and *L. elongisporus* lack the *EST1* gene, and their Est3 proteins contain large N- and C-terminal extensions, which are important for the interaction with TEN domain in vitro^60^. One might speculate that these extensions evolved to strengthen the Est3-TEN bond, thereby compensating for the Est1 loss^62^. Thus, it is possible that Est3-TEN and Est3-Est1 interactions are both able to provide a robust recruitment mechanism for Est3, however different yeast species evolved to rely more on one particular interaction (like *H. polymorpha*, or *C. parapsilosis* and *L. elongisporus*), or to utilize both of them (like in case of *S. cerevisiae*).

We uncovered a substantial reduction of tagged TERT in *H. polymorpha* cells lacking either Est1 or Est3. At least in *∆est3* cells, this reduction is at the protein level, suggesting that either TERT mRNA translation or protein stability are affected. Est3 (as well as Est1) has not been implicated in translation regulation; therefore, its effect on the TERT stability is more plausible. Based on the results of this study, we propose that the combined action of both Est1 and Est3 promotes productive association of TERT with telomerase RNA in *H. polymorpha*. Following the loss of any partner of a pair Est1/Est3, the second partner can no longer form a stable complex with TER. In the absence of the Est1/Est3 pair, TERT-TER interaction weakens, leading to the increase of free TERT, which is presumably more susceptible to degradation by cellular proteases than within a complex containing TER. Consistent with this idea, HpTER deletion also dramatically diminishes the cellular pool of TERT-HA.

In *S. cerevisiae*, it has been shown that stable association of Est1 with TER in vivo requires the presence of the Pop1/Pop6/Pop7 proteins pre-bound near the Est1 RNA-binding site^22,23^. The regions of TER binding Est1 and the Pop proteins appear to be present in other yeast species, including *H. polymoprha*^22,49^. It is possible that HpEst3 binds HpEst1 and the homologues of the Pop proteins in *H. polymorpha*, which could explain the positive effect of Est3 on Est1-TER binding observed in our experiments.

Telomerase is an evolutionarily variable enzyme. An active in vivo telomerase complex includes a large number of protein components^63^. Their number and structure is very different for various organisms. Only two components (TER and TERT) are indispensable, although their structure is variable. Other accessory proteins are diverse in both structure and function. For instance, in contrast to yeast Est3, the human analogue hTPP1 has two additional domains to the C-terminus of the OB-domain: the POT1-binding domain and the TIN2-binding domain^64^. POT1 protein interacts directly with the single-stranded telomeric DNA and maintains the integrity of telomeres^65^. hTPP1 alone doesn’t bind ssDNA, but the complex of POT1 with hTPP1 exhibits a tenfold increase in the affinity toward ssDNA^44,57^. Analysis of available structures of yeast Est1 from *Kluyveromyces lactis* (KlEst1)^66^ and human Est1 homologues SMG5, SMG6, and SMG7^67,68^ revealed high structural similarity of the Est1 TPR domain with a family of 14-3-3 proteins. The latter are known to act as hub-proteins and mediate numerous protein–protein interactions via binding to the unstructured fragments of their partners^69^. It is worth noting that HpEst3 also has a long disordered tail, which can be involved in the binding to HpEst1 in a 14-3-3-like fashion.

## Conclusions

The high-resolution structure of HpEst3 in solution has been determined. This is a first structure of the Est3 protein determined with a large number of experimental restraints. The dynamic properties of HpEst3 were studied, indicating that the protein has a conformational mobility of its core, typical for proteins involved in protein–RNA and/or protein–protein interactions. NMR titration experiments indicate that free Est3 does not interact specifically with either the N-terminal domain of TERT or with DNA and/or RNA fragments mimicking the probable telomerase environment. It was found, however, that both Est3 and Est1 are essential for the formation of stable and functionally active telomerase in *Hansenula polymorpha*.

## Materials and methods

### Yeast strains

Strains used in this study are listed in Supplementary Table S2. Oligonucleotides used for PCR during strain construction are listed in Supplementary Table S3. The DL1-L strain^70^ was used as a wild type (no tag) control in all experiments. Gene replacements were performed by transformation of the DLdaduA^71^ or another appropriate strain with DNA integration cassettes according to a standard protocol, modified as described^72^. For *EST3* gene knock-out, EST3 ORF with flanking regions (PCR product#1) was cloned in pUC19; NheI/XhoI fragment of the resulting plasmid was then substituted with the SalI/XbaI fragment of the pCHLX vector^73^. *TERT* and *EST1* gene knock-outs were performed as described previously^48,49^. For C-terminal HA-tagging we used a pFA6a-3HA-HpURA3 plasmid^74^. PCR products #2 and #3 were cloned at the SalI/XmaI and PmeI/ClaI sites (respectively) of the pFA6a-3HA-HpURA3 vector for construction of the *TERT-HA* strain; PCR products #4 and #5—for the EST3-HA strain; PCR products #6 and #7—for the *EST1-HA* strain. Correct integration of the cassettes and gene replacements were verified by PCR.

### Spot assay

Several *∆est3* colonies after transformation were grown overnight (“passage 1”) in 2 ml of complete minimal medium^75^ without leucine (SC-LEU); 1 ml of culture was used for genotype verification. The rest was inoculated in 100 ml of SC-LEU (OD_600_ ~ 0.05) and grown overnight at 37 °C (“passage 2”). Two independent *∆est3* transformants were passaged six more times. Each time, 10 μl aliquots of cultures with OD_600_ ~ 0.05 (along with three tenfold dilutions) were spotted onto YPD (1% yeast extract, 2% peptone, 2% glucose) agar plate and grown for 2 days at 37 °C. 50 ml of the overnight cultures during each passage were collected and used for telomere length measurements.

### Telomere Southern blots

PstI-digested genomic DNA was separated on 1% agarose gel, then transferred to a nylon membrane (Whatman Nytran SuPerCharge). Southern hybridization was performed according to the standard protocol ^76^. 5′-radiolabelled C4 oligonucleotide (5′-(CGCCACCC)_4_-3′) was used as a probe.

### Immunoprecipitation (IP) experiments

Typically, yeast cells were grown to OD_600_ ~ 1 in 400 ml of YPD (1% yeast extract, 2% peptone, 2% glucose) medium at 37 °C. S In case of telomerase null cells, we used only freshly prepared knock-outs for IP experiments. Several colonies after transformation were grown overnight in 2 ml of SC-LEU: 1 ml of culture was used for genotype verification, and to another 1 ml portion we added 2 ml of YPD and grew overnight. Then, these cultures were diluted in 400 ml of YPD and grown to OD_600_ ~ 1.

Cells were collected by centrifugation, resuspended in 1 ml of binding buffer [20 mM HEPES-NaOH pH 7.5, 2 mM MgCl_2_, 10% glycerol, 0.1% Nonidet P-40, 150 mM NaCl, 1 mM DTT, Halt Protease and Phosphatase Inhibitor Cocktail (Thermo Scientific)] and broken by glass beads in Precellys Evolution homogenizer. Cell extracts were cleared by centrifugation (16,000*g*, 20 min). 1 ml of lysates were incubated with 40 μl (1:1 suspension) of anti-HA agarose (Roche, A2095) at + 4 °C for 1 h. Beads were washed three times with binding buffer. Half of the beads were analyzed by Western blot and the other half by qRT-PCR.

For experiments shown in Fig. 2c, d, 1 ml of *TERT-HA EST3* lysate and 4 ml of *TERT-HA ∆est3* lysate (prepared from 400 ml and 1,600 ml of YPD cultures, respectively) were incubated with 40 μl of anti-HA beads. For Western blot we used 1/10 (*TERT-HA EST3*) and 1/2 (*TERT-HA ∆est3*) of beads; for telomerase activity assay—1/20 (*TERT-HA EST3*) and 1/4 (*TERT-HA ∆est3*) of beads. All IP experiments were performed in triplicate.

### Western blot analysis

Proteins were eluted from anti-HA resin by incubation with 15 μl of HU buffer (7 M urea, 5% SDS, 0.2 M Tris–HCl pH 6.8, 1 mM EDTA, 0.2% bromophenol blue) at 95 °C for 10 min. Eluates or input samples were separated by 6% (*TERT-HA* and *Est1-HA*) or 12% (*Est3-HA*) SDS-PAGE. Anti-HA-HRP antibodies (clone 3F10, Sigma-Aldrich) at 1:2000 and a subsequent Western Bright ECL Kit (Advansta) were used for detection. The linearity of the TERT-HA signal was verified by analyzing serial two-fold dilutions of IP samples and extracts containing TERT-HA.

### qRT-PCR

½ portions of anti-HA beads or 3 μl aliquots of extracts (0.3% of input) were diluted in a binding buffer to a final volume 100 μl, supplemented with SDS (0.1% final); then treated with 40 μg of proteinase K at 37 °C for 30 min. RNA was extracted with phenol/chloroform, precipitated with EtOH and dissolved in 20 μl of mQ water. Samples were treated with DNase I (Thermo Fisher Scientific). cDNA synthesis and PCR reactions were performed as described^49^, with the only modification being that we used Maxima RT (Thermo Fisher Scientific) for cDNA production. Data was represented as yields (% of input), the values calculated using the formula: 2^(Ct(input) − Ct(beads)) × 2 × 0.3.

### Telomerase activity assay

50 μl mixtures [containing 15 μl anti-HA beads (resuspended in binding buffer without Nonidet P-40), 1 μM HD5 oligonucleotide (5′-AAAAAGGGTGGCG-3′), 50 mM Tris–HCl pH 8, 1 mM spermidine, 1 mM DTT, dATP/dTTP/dCTP (50 μM each), and 3.75 μM α-^32^P-dGTP (800 Ci/mmol)] were incubated at 30 °C for 30 min. Reactions were stopped by proteinase K treatment, extracted with phenol/chloroform and precipitated with EtOH. 5′-^32^P-labeled oligonucleotide 5′-AAAAAAGGGTGGC-3′ was added after proteinase K treatment served as a loading control. Products were resolved on 10% denaturing PAGE.

### Expression of HpEst3 and HpTEN

HpEst3 and HpTEN were expressed and purified as described in Refs.^47^^,^^77,78^ correspondingly.

### RNA and DNA synthesis

DNA and RNA oligonucleotides were assembled in an MM-12 synthesizer (Bioautomation) with the phosphoramidite method, according to the manufacturer's recommendations at 25 µmol scale. Synthetic procedure is described in detail earlier^78^.

### NMR spectroscopy

The NMR samples in concentration of 0.4 mM for ^13^C,^15^N-labeled HpEst3 and 0.2–0.4 mM for ^15^N-labeled protein were prepared in 90% H_2_O/10% D_2_O, 100 mM KCl, and 20 mM potassium phosphate buffer (pH 6.5). DTT in concentration of 3 mM was added to the final solution to prevent oxidation of three cysteine residues (C29, C61 and C63). Triple-resonance (^1^H,^13^C,^15^N) spectra were acquired at 298 K on a Bruker Avance III HD 700 MHz spectrometer equipped with a quadruple resonance (^1^H,^13^C,^15^N,^31^P) QCI CryoProbe. ^15^N-^1^H HSQC and SOFAST HMQC spectra on ^15^N-labeled HpEst3 in NMR titration experiments were acquired at 298 K on a Bruker Avance 600 MHz spectrometer equipped with a triple resonance (^1^H,^13^C,^15^N) TXI probe. All spectra were processed by NMRPipe^79^, and analyzed using NMRFAM-SPARKY^80^.

### NMR structure determination

The family of 20 NMR structures of HpEst3 was calculated using conformational restraints for 2,262 inter-proton distances and 228 backbone dihedral angles (Table 1). A set of dihedral angles was obtained from the analysis of the ^1^HN, ^1^Hα, ^15^N, ^13^Cα, ^13^Cβ and ^13^C′ chemical shifts using the TALOS + software ^81^ for the residues located in the well-ordered regions of the protein core, as defined by NMR relaxation experiments and the RCI (Random Coil Index) approach^82^. NOEs, used as distance restraints in structure calculation, were obtained from the analysis of cross-peak intensities in the 3D ^13^C-^1^H and ^15^N-^1^H HSQC-NOESY spectra. The intra-residue and sequential cross-peaks of NOESY spectra were assigned manually, while the rest of the cross-peaks were assigned using the automatic iterative procedure of spectra assignment/structure calculation implemented in ARIA 2.3 software^83^. The automatic assignment and the inter-proton distances provided at the last iteration of the ARIA 2.3 protocol were further manually verified by multiple steps of the structure refinement accomplished using the simulated annealing protocol of the CNS 1.21 software package^84^. Structure refinement included a high-temperature torsion-angle molecular dynamics stage followed by a slow-cooling torsion-angle phase, a second slow-cooling phase in Cartesian space and Powell energy minimization. Database values of conformational torsion angle pseudopotentials^85^ were implemented during the final cycles of the calculations to improve the quality of protein backbone conformation. Structure refinement was performed until no NOE violations larger than 0.5 Å and no dihedral angle violations higher than 5° occurred. The restraint violations and structure quality were assessed using the CNS tools, Procheck-NMR^86^, and in-house software and utilities. At the last iteration of the refinement protocol 200 structures were calculated using 2,262 unambiguous distance and 228 dihedral angle restraints. The final family of 20 NMR structures was filtered out in accordance with the lowest-energy criterion. Statistics for the determined NMR structures are presented in Table 1. Structure visualization and analysis were carried out using PyMOL (Schrödinger LLC).

### NMR dynamics analysis

R_1_, R_2_ and ^1^H-^15^N heteronuclear NOE data sets of ^15^N uniformly labeled HpEst3 were collected at 298 K on a Bruker Avance III HD 700 MHz spectrometer. The delays for the R_1_ relaxation rate experiments were 0.2, 0.25, 0.3, 0.35, 0.4, 0.45, 0.5, 0.58, 0.64, 0.8, 1.0, 1.3, 1.8, 2.5 s; and for the R_2_ relaxation rate experiments were 0, 17, 33.9, 50.9, 67.8, 84.8, 101.8, 118.7, 135.7, 152.6, 169.6, 186.6, 203.5, 237.4, 271.4, 305.3 ms. The excitation time for ^1^H in the ^1^H-^15^N heteronuclear NOE experiments was 4.0 s. Spectra were processed using NMRPipe software^79^. The nonlinear fitting of the integrated peak volumes in the pseudo 3D spectra of the relaxation experiments and the calculation of standard deviations were accomplished using the nlinLS procedure. The values of R_1_ and R_2_ were then calculated from the table of relative peak intensities, produced by NMRPipe and nlinLS, using RelaxFit, which was written in-house^87^. The standard deviations of the ^15^N-^1^H NOE values were calculated using the RMS noise of the background regions^88^ and were further checked and corrected using two independently collected experimental data sets. The analysis of the R_1_, R_2_ and ^1^H,^15^N-NOE values was carried out using a model-free formalism using the RelaxFit program^87^. To determine the rotational diffusion tensor, all of the isotropic, axially symmetric, and fully asymmetric molecular tumbling models were tested. The values of the correlation time of protein tumbling or the diffusion tensor axis were then used to fit models of internal motions for the backbone HN vectors of the amino acid residues.

### NMR titration experiments

Protein–protein and protein-nucleic acids interactions were tested using the ^15^N,^1^H SOFAST HMQC experiments measured at 25 °C and 600 MHz ^1^H frequency. A single ^15^N-labelled protein (HpEst3 or HpTEN, concentration between 0.15 and 0.40 mM) was used to monitor intermolecular interactions, while one or more unlabelled substances were gradually added to the sample. Reaction mixtures were studied in a buffer which contained 100 mM KCl, 20 mM potassium phosphate (pH = 6.5), 0.02% NaN_3_, and 3 mM DTT. The pH of the unlabelled protein samples was adjusted by dialysis against this buffer. These samples were then aliquoted and freeze-dried to prevent a change in the concentration of the ^15^N-labeled protein during the titration. Short unlabelled oligonucleotides (see Supplementary Table S1) were used to examine protein-NA interactions. The pH values of the oligonucleotides were adjusted to be identical to the pH of ^15^N-labelled protein solution, and then aliquoted and freeze-dried. DNA-RNA heteroduplexes were prepared using the annealing protocol, described earlier^78^. The RNA hairpin was prepared at 70 °C and low concentration of RNA (50 μM) to avoid oligomerisation. A RiboLock RNase inhibitor (Thermo Fisher Scientific), in concentration of 700 U/ml was added to the samples containing the single-stranded RNA in order to inhibit RNA cleavage. The protocol for carrying out NMR titration experiments is generally identical to that previously described by us for the HpTEN^78^.

### Electrophoretic mobility shift assay (EMSA)

EMSA was performed essentially as described earlier^74^ the following modifications. 1 μM fG4 oligonucleotide (FAM-5′-(GGGTGGCG)_4_) was incubated with an increasing amount of Est3 (concentration range: 0, 1, 3, 10 μM). Reactions were performed in a solution of 20 mM potassium phosphate (pH = 6.5), 100 mM KCl, and 3 mM DTT. Products were separated in an 8% non-denaturing polyacrylamide gel (19:1).

### Equipment and settings for gel/blot images

Gels and blots from this study were acquired as described earlier^74^ with the following modifications. The Western blot images were acquired using the “Chemi Hi Resolution” application. The gels from the telomerase activity assays were acquired on the Typhoon FLA 7000 (GE Healthcare) imaging system using the “Phosphor” method. Processing (cropping and contrast adjustments) was performed in ImageLab 5.2.1 software, ImageQuant TL 7.0 or Adobe Photoshop CC 2018. Contrast adjustments were applied equally across the entire images (including controls).

## Supplementary information

Supplementary file1 (PDF 2701 kb)

## Data Availability

The structural data and experimental restraints used in calculations have been submitted to the Protein Data Bank with accession number 6Q44.

## Abbreviations

CaEst1, CaEst3, CaTER: Est1, Est3 proteins and TER from *Candida albicans*
HpTER, HpTERT, HpEst1, HpEst3: TER, TERT, Est1 or Est3 from *Hansenula polymorpha*
hPOT1, hTPP1: Human proteins POT1, TPP1
EMSA: Electrophoretic mobility shift assay
HSQC: Heteronuclear single quantum coherence spectroscopy
NOE: Nuclear Overhauser effect
NOESY: Nuclear Overhauser enhancement spectroscopy
*R*_1_: Longitudinal or spin–lattice relaxation rate
*R*_2_: Transverse or spin–spin relaxation rate
*R*_*ex*_: Conformational exchange contribution to *R*_2_
RMSD: Root-mean-square deviation
*S*^2^: Order parameter reflecting the amplitude of ps-ns bond vector dynamics
ScEst3, ScTERT: Est3 or TERT proteins from *Saccharomyces cerevisiae*
SnTEBP: TEBP protein from *Sterkiella nova*
SOFAST HMQC: Band-selective optimized flip angle short transient heteronuclear multiple-quantum correlation experiment
TEN: N-terminal domain of TERT
TERT: Telomerase reverse transcriptase
TER: Telomerase RNA
*TERT, EST1, EST3*: Genes encoding TERT, Est1, Est3 proteins
TERT-HA, Est1-HA, Est3-HA: TERT, Est1 or Est3 proteins tagged with a hemagglutinin epitope (HA)
*TERT-HA*, *EST1-HA*, *EST3-HA*: Strains expressing TERT (Est1, Est3) tagged with a hemagglutinin epitope
*τ*_*e*_: Effective internal correlation time
*τ*_*m*_: Overall rotational correlation time
∆*tert, ∆est1, ∆est3*: Strains with *TERT* (*EST1*,* EST3*) gene deleted
