# Supplementary material for: Insights into the structure and function of Est3 from the Hansenula polymorpha telomerase
Source: Sci Rep. 2020 Jul 6;10:11109. doi: 10.1038/s41598-020-68107-x (PMC7338525; doi:10.1038/s41598-020-68107-x)
Supplement: Supplementary file 1 — Supplementary file1 (PDF 2701 kb) [file 41598_2020_68107_MOESM1_ESM.pdf]

Supplementary Data

Insights into the structure and function of Est3 from the *Hansenula polymorpha* telomerase

Nikita Shepelev, Sofia S. Mariasina, Alexey B. Mantsyzov, Alexander N. Malyavko, Sergey V. Efimov, Olga A. Petrova, Elena V. Rodina, Maria I. Zvereva, Olga A. Dontsova and Vladimir I. Polshakov

Contents	Page number
**Table S1**. NMR titration experiments to probe interactions between Est3 *Hansenula polymorpha* and its putative partners in telomerase.	S2
**Table S2**. *H. polymorpha* strains used in this study.	S3
**Table S3**. List of PCR products used during construction of *H. polymorpha* strains.	S4
**Figure S1**. NOE histogram for each protein residue in the HpEst3.	S5
**Figure S2**. Ramachandran plot for the family of 20 HpEst3 conformers.	S6
**Figure S3**. Fragments of ^{15}N-^{1}H spectra of HpEst3 recorded at different temperatures in the range between 15 and 30°C.	S7
**Figure S4**. Titration of the ^{15}N-labelled HpEst3 by the unlabeled TEN domain.	S8
**Figure S5**. Titration of the ^{15}N-labelled HpEst3 by the ssDNA fragment GTGGCGGGGTGGCG.	S9
**Figure S6**. Titration of the ^{15}N-labelled HpEst3 by the ssDNA fragment GGGTGGCGGGGTGGCGGGGTGGCGGGGTGGCG.	S10
**Figure S7**. Titration of the ^{15}N-labelled HpEst3 by the ssDNA fragment GTGGCGGGGTGGCG in presence of the unlabeled TEN domain.	S11
**Figure S8**. Titration of the ^{15}N-labelled TEN domain by the unlabeled HpEst3.	S12
**Figure S9**. Titration of the ^{15}N-labelled TEN domain by the ssDNA GTGGCGGGGTGGCG in presence of the unlabeled HpEst3.	S13
**Figure S10**. Titration of the ^{15}N-labelled TEN domain by the RNA hairpin.	S14
**Figure S11**. Titration of the ^{15}N-labelled TEN domain by the RNA-DNA heteroduplex.	S15
**Figure S12.** Electrophoretic mobility shift assay experiment with HpEst3.	S20
**Figure S13.** Quantitative RT-PCR analysis of the HpTER co-precipitated on anti-HA agarose and results of telomerase activity analysis.	S16
**Figure S14.** Original gels/blots for Figure 1.	S17
**Figure S15.** Original gel/blots for Figures 1 and 2.	S18
**Figure S16.** Original gel/blots for Figure 6.	S19

Table S1. NMR titration experiments to probe interactions between Est3 *Hansenula polymorpha* and its putative partners in telomerase. All experiments were carried out in the buffer solution containing 100 мM KCl, 20 мM potassium phosphate (pH 6.5), 0.02% NaN_3, and 3 mM DTT, at 298 K.

#	^{15}N-labeled target	Additional compo-nents	Unlabeled titrant	Final mixture composition
1	HpEst3	-	HpTEN	Est3:TEN = 1 : 7
2	HpEst3	-	ssDNA 1.8<br>5′-GTGGCGGGGTGGCG-3′	Est3:DNA = 1 : 4.5
3	HpEst3	-	ssDNA G4<br>5′-GGGTGGCGGGGTGGCGGGGTGGCGGGGTGGCG-3′	Est3:DNA = 1 : 5
4	HpEst3	ssDNA 1.8	HpTEN	Est3:DNA:TEN = 1:4.5:1.5
5	TEN	-	HpEst3	TEN:Est3 = 1 : 3.5
6	TEN	HpEst3	ssDNA 1.8<br>5′-GTGGCGGGGTGGCG-3′	TEN:Est3:ssDNA = 1 : 3.5 : 3.0
7	HpEst3	TEN	RNA upstream (RNAup)<br>5′-UUCGUCA-3′	Est3:TEN:RNA = 1:4:4
8	HpEst3	DNA1.8<br>TEN	RNA hairpin (RNAhp)<br>A<br>5'**AACAAAUGGAGACG** U<br>3'**UUGUGUACCUCUGC** C<br>C	Est3:ssDNA:TEN:RNAhp = 1 : 4.5 : 3 : 8
9	HpEst3	DNA 1.8<br>TEN<br>RNAhp	RNA-DNA heteroduplex<br>DNA: 3'GCGGTGGGGCGGTG5'<br>RNA: 5'CGCCACCCCGCCAC3'	Est3:ssDNA:TEN:RNAhp: heteroduplex = 1 : 4.5 : 3 : 8 : 2.5
10	HpEst3	TEN	RNA-DNA fork<br>DNA: 5'CTGTTTC**GGGTGGC**3'<br>RNA: 3'ACUGCUU**CCCACCG**5'	Est3:TEN:fork = 1 : 1 : 4
11	HpEst3		3',5'-cGMP	Est3:cGMP = 1 : 10

Table S2. *Hansenula polymorpha* strains used in this study.

Strain name	Genotype	Reference
DL1-L	DL-1 (ATCC 26012) *leu2*	(1)
DLdaduA	DL-1 (ATCC 26012) *leu2 Δade2 Δura3::ADE2*	(2)
Δest3	DL-1 (ATCC 26012) *leu2 Δest3::HpLEU2*	This study
TERT-HA	DL-1 (ATCC 26012) *leu2 Δade2 Δura3::ADE2 TERT-3HA::HpURA3*	This study
TERT-HA Δest3	DL-1 (ATCC 26012) *leu2 Δade2 Δura3::ADE2 TERT-3HA::HpURA3 Δest3::HpLEU2*	This study
TERT-HA Δest1	DL-1 (ATCC 26012) *leu2 Δade2 Δura3::ADE2 TERT-3HA::HpURA3 est1::HpLEU2*	This study
EST3-HA	DL-1 (ATCC 26012) *leu2 Δade2 Δura3::ADE2 EST3-3HA::HpURA3*	This study
EST3-HA Δtert	DL-1 (ATCC 26012) *leu2 Δade2 Δura3::ADE2 EST3-3HA::HpURA3 tert::HpLEU2*	This study
EST3-HA Δest1	DL-1 (ATCC 26012) *leu2 Δade2 Δura3::ADE2 EST3-3HA::HpURA3 est1::HpLEU2*	This study
EST1-HA	DL-1 (ATCC 26012) *leu2 Δade2 Δura3::ADE2 EST1-3HA::HpURA3*	This study
EST1-HA Δtert	DL-1 (ATCC 26012) *leu2 Δade2 Δura3::ADE2 EST1-3HA::HpURA3 tert::HpLEU2*	This study
EST1-HA Δest3	DL-1 (ATCC 26012) *leu2 Δade2 Δura3::ADE2 EST3-3HA::HpURA3 Δest3::HpLEU2*	This study

(1) Kang, H. A., Sohn, J.-H., Agaphonov, M. O., Choi, E.-S., Ter-Avanesyan, M. D. & Rhee, S. K. (2002). Development of expression systems for the production of recombinant proteins in *Hansenula polymorpha* DL-1. In *Hansenula polymorpha* (Gellissen, G., ed.), pp. 124-146. Wiley-VCH Verlag GmbH & Co. KGaA. DOI: 10.1002/3527602356

(2) Karginov, A. V., Fokina, A. V., Kang, H. A., Kalebina, T. S., Sabirzyanova, T. A., Ter-Avanesyan, M. D. & Agaphonov, M. O. (2018). Dissection of differential vanadate sensitivity in two *Ogataea* species links protein glycosylation and phosphate transport regulation. *Sci. Rep.* **8**, 16428. DOI: 10.1038/s41598-018-34888-5.

Table S3. List of PCR products used during construction of *Hansenula polymorpha* strains.

PCR product #	Product description	Primer sequence (5’ – 3’)
1	EST3 gene	F: GTGAAGGCAACGAACGATGG
		R: CGGCTAAAGGAGATGCAGAC
2	5’ part of the cassette for TERT-HA strain	F: AAAAGTCGACTACCTGCTCTCGCTAGGAGG
		R: AAACCCGGGGAAAGTTTTGAGAAGCGAACGGATAAAG
3	3’ part of the cassette for TERT-HA strain	F: AAAAGTTTAAACAGCATTAAAAGTCTCGTTTCTAATCAC
		R: AAATCGATATGTCCATCGAACGCAAGAACTG
4	5’ part of the cassette for EST3-HA strain	F: AAAAGTCGACTGCCAGTGCCCACCATGAAG
		R: AAACCCGGGTTCTTCATCACTAAATTCCTGCTCTATAAAC
5	3’ part of the cassette for EST3-HA strain	F: AAAAGTTTAAACGATCCCCTCAAAATTCCTGAGG
		R: AAATCGATCGGCTAAAGGAGATGCAGACAC
6	5’ part of the cassette for EST1-HA strain	F: AAAAGTCGACTCAATTGTAAGCGATCCC
		R: AAACCCGGGTTTTCCATATTGGTGATACGC
7	3’ part of the cassette for EST1-HA strain	F: AAAAGTTTAAACGACTTTTTATATTGAAAGAGTCGGTTGC
		R: AAATCGATGACTACGCTATCCGACATAGATCC

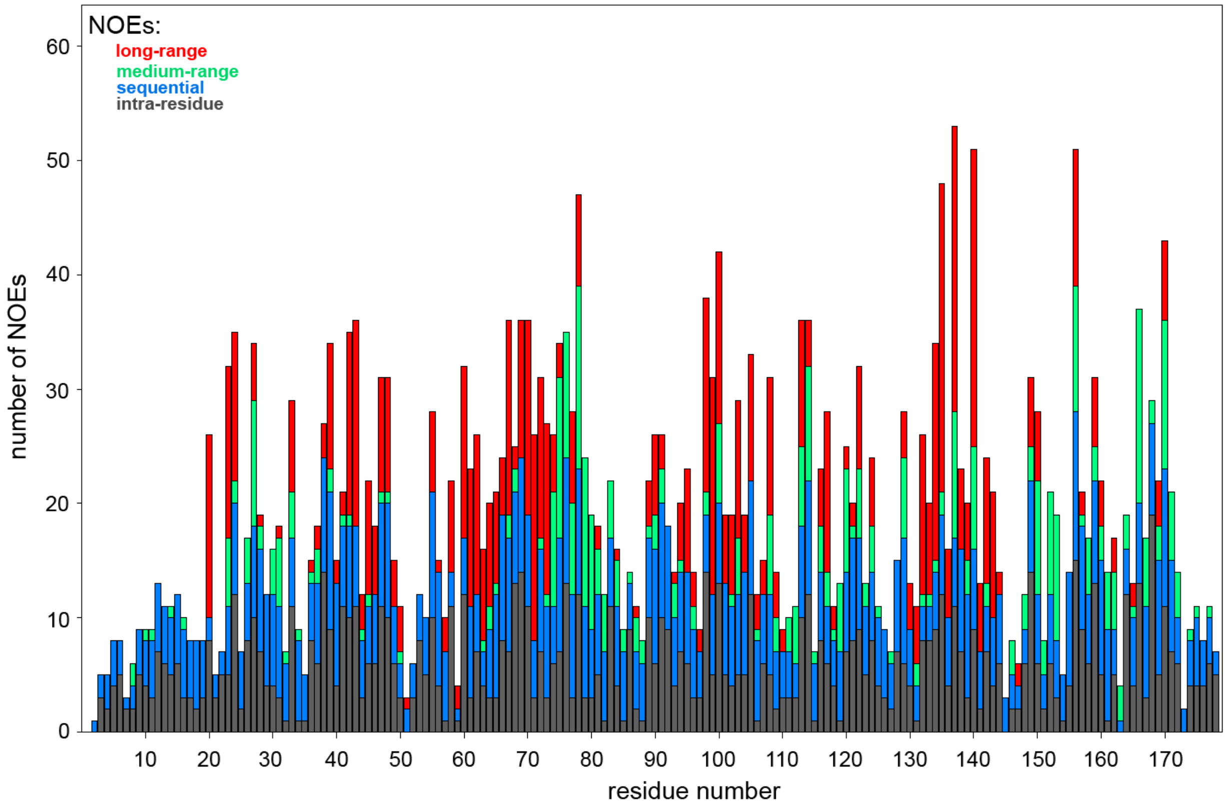

Figure S1. NOE histogram giving the number of long-range (red), medium-range (green), sequential (blue) and intra-residue (dark grey) NOEs for each protein residue in the HpEst3.

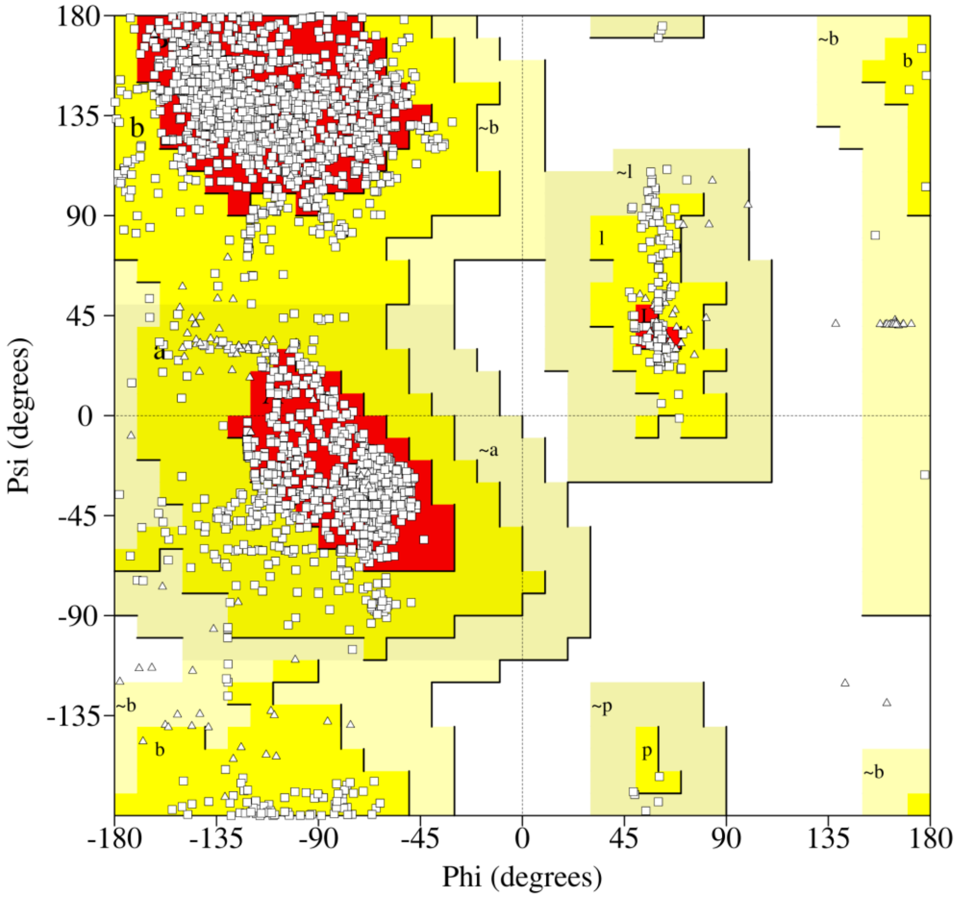

Figure S2. Ramachandran plot for the family of 20 HpEst3 conformers. No residues fall in disallowed regions, 87% of residues fall in the most favorable regions. Most of the residues in the generously allowed regions are from the unstructured N- and C-terminal tails of the protein.

Figure S3. Fragments of ^{15}N-^{1}H spectra of HpEst3 recorded at different temperatures in the range between 15 and 30°C.

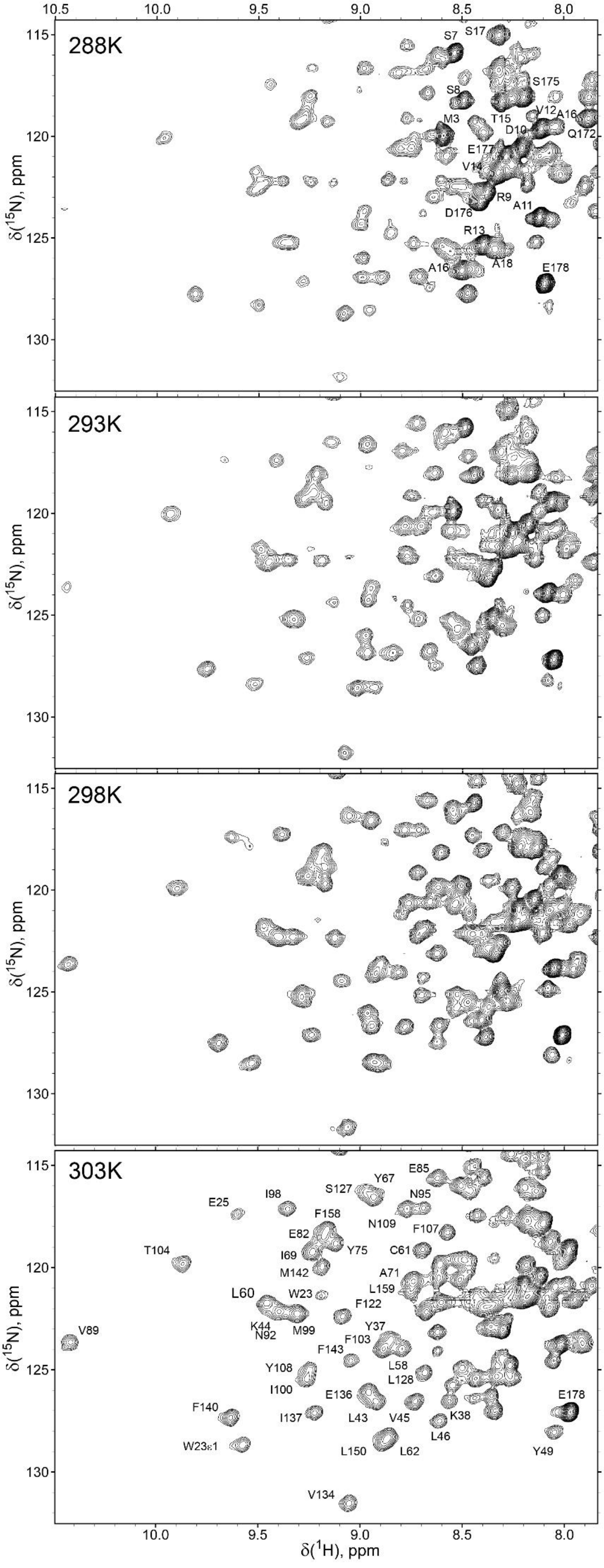

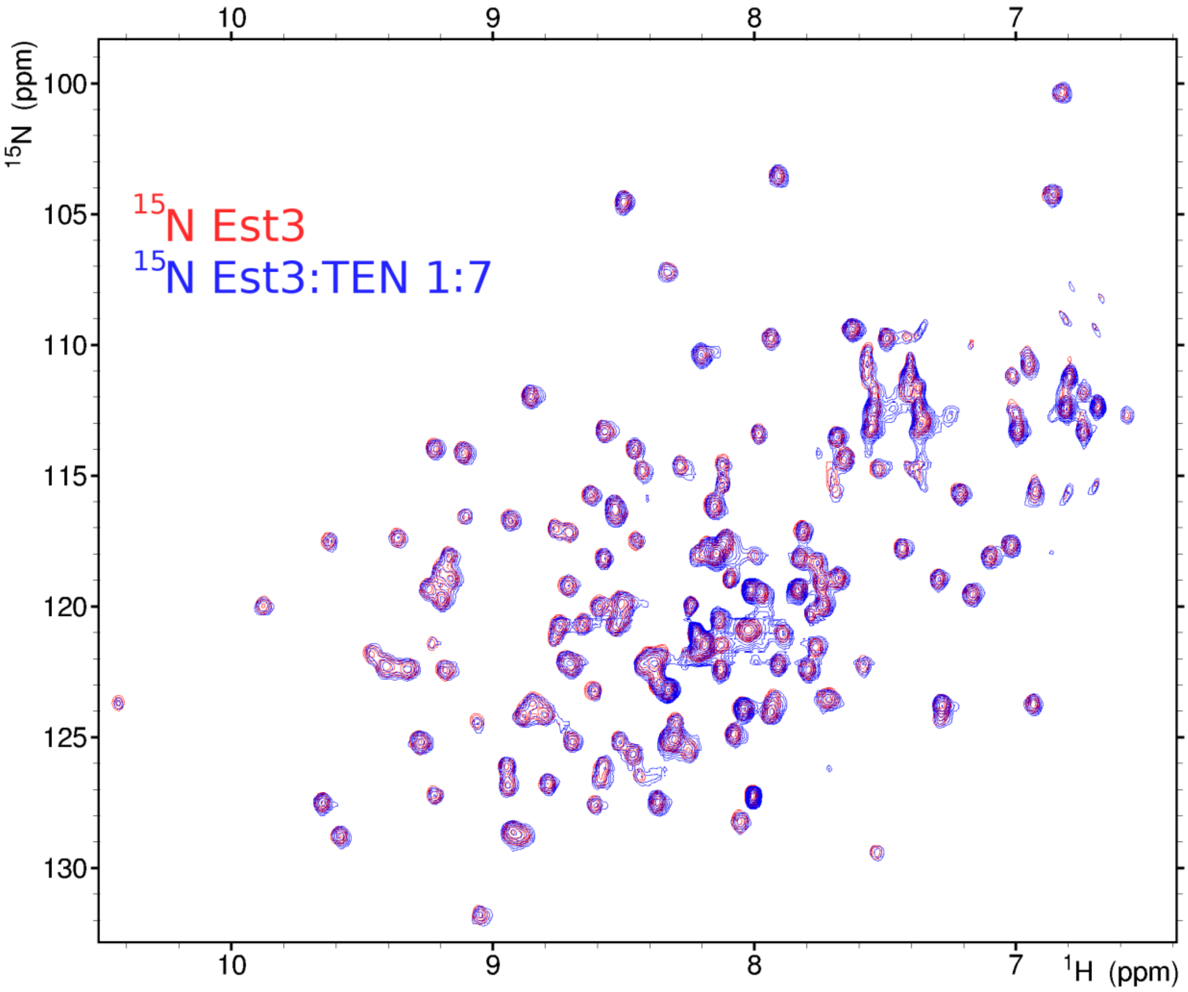

Figure S4. Titration of the ^{15}N-labelled HpEst3 by the unlabeled TEN domain.

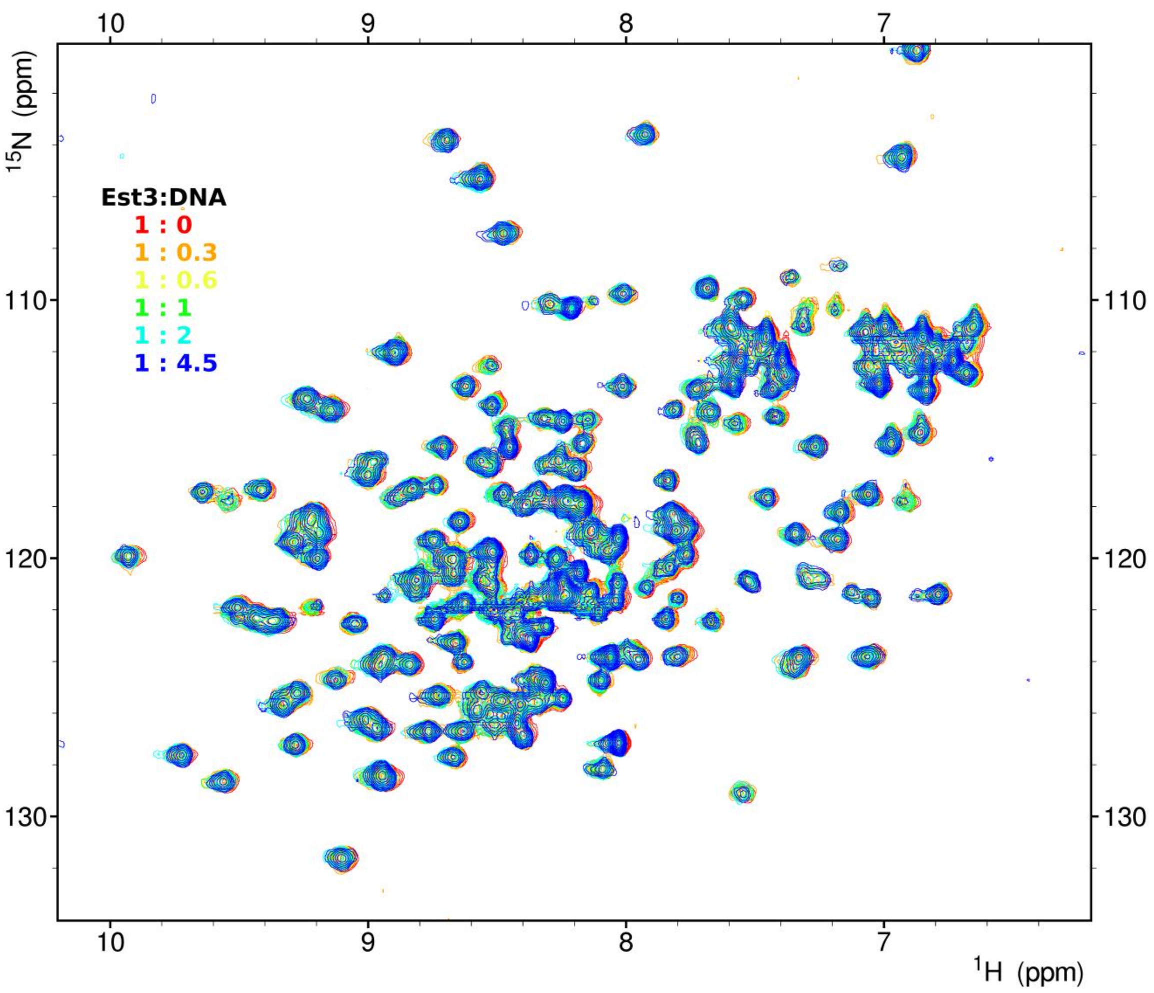

Figure S5. Titration of the ^{15}N-labelled HpEst3 by the ssDNA fragment 5'-GTGGCGGGGTGGCG-3'.

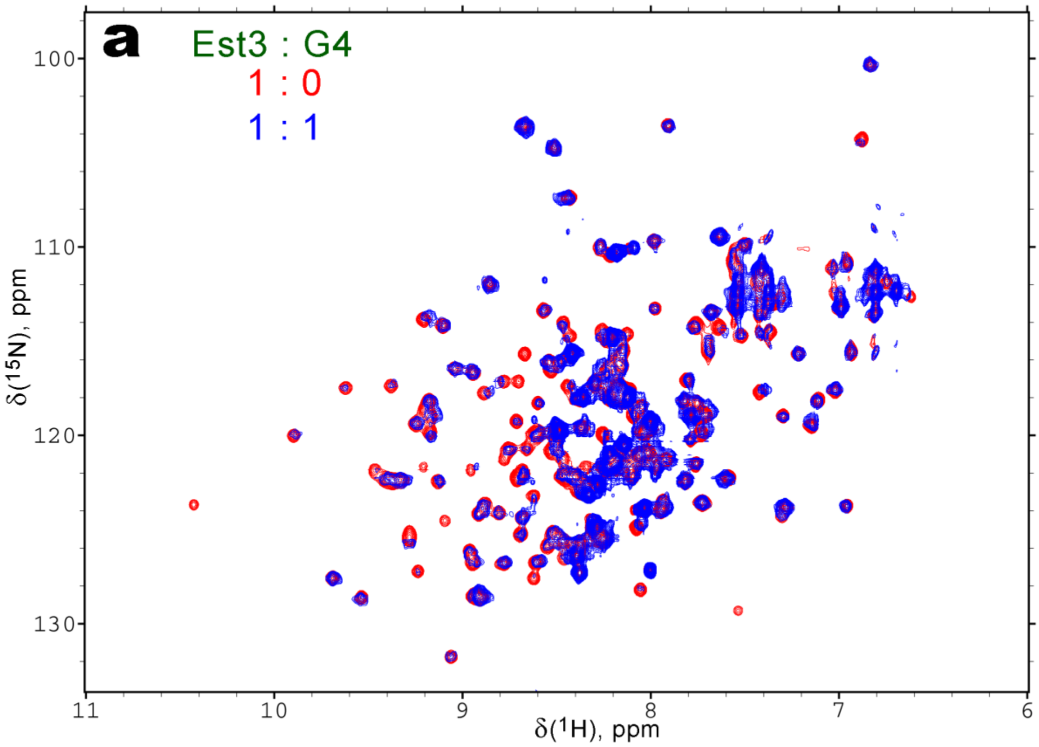

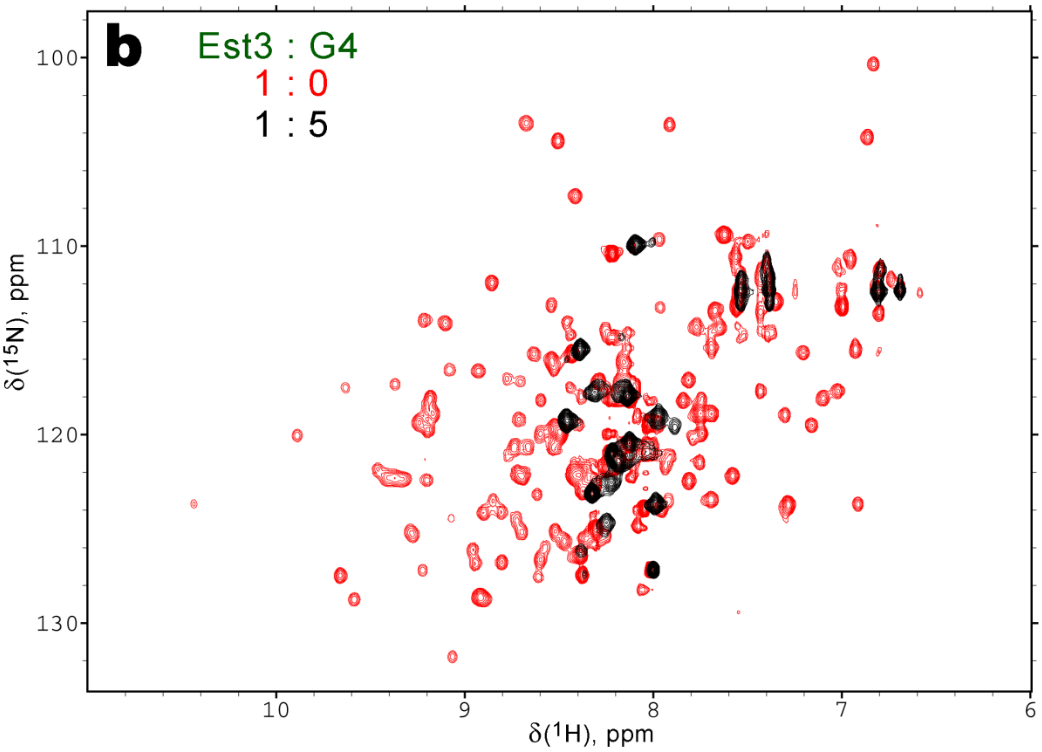

Figure S6. Titration of the ^{15}N-labelled HpEst3 by the ssDNA fragment G4 (5'-GGGTGGCGGGGTGGCGGGGTGGCGGGGTGGCG-3'). **A**. Overlay of the ^{15}N-^{1}H SOFAST-HMQC spectra of ^{15}N-HpEst3 in the absence (red) and in presence (blue) of 1 molar equivalent of G4. **B**. Overlay of the ^{15}N-^{1}H SOFAST-HMQC spectra of ^{15}N-HpEst3 in the absence (red) and in presence (black) of 5 molar equivalents of G4. Only the signals of the residues from the unstructured HpEst3 tail and side-chain amide groups remain in second spectrum. Other resonances are not seen due to their large line width.

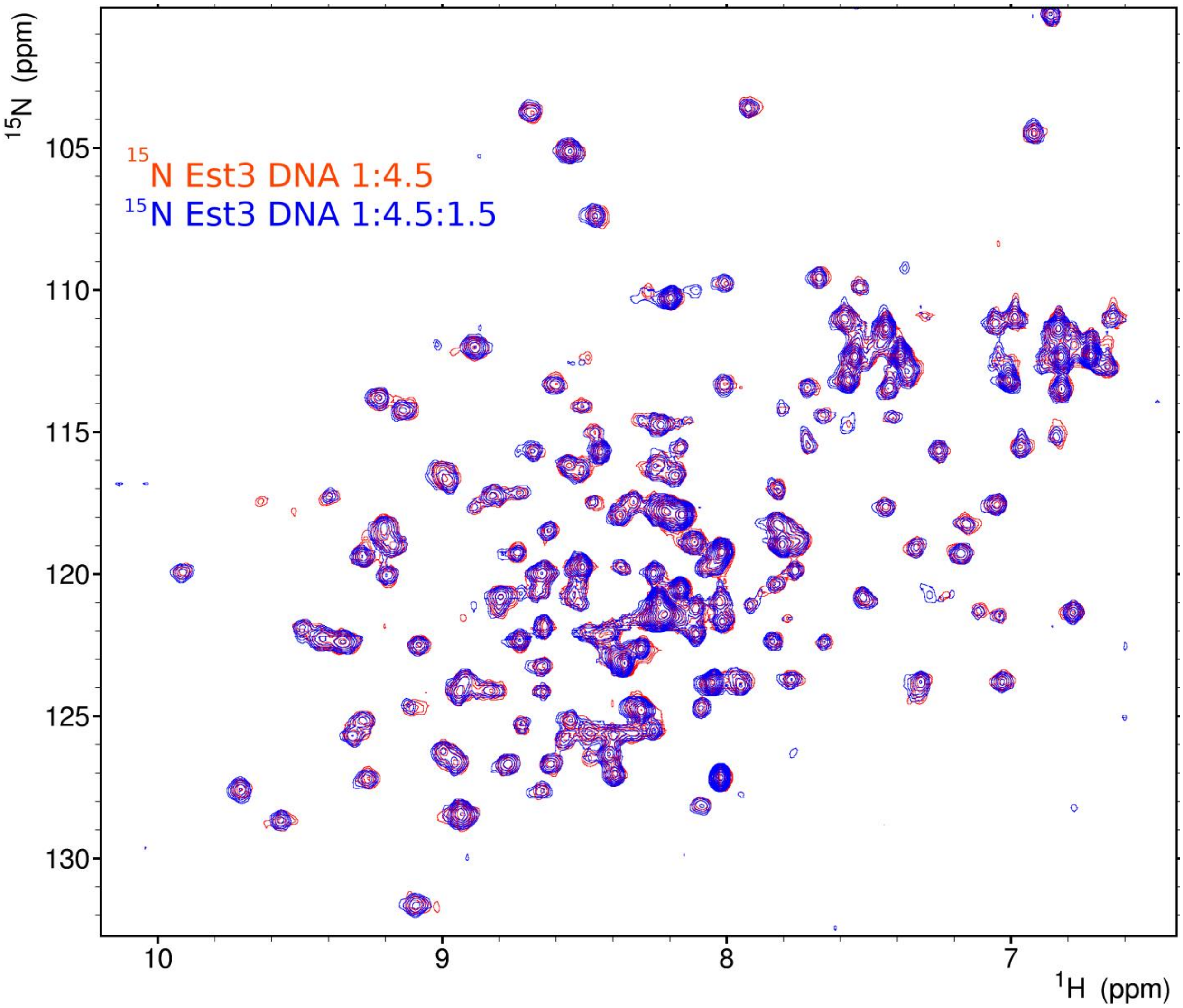

Figure S7. Titration of the ^{15}N-labelled HpEst3 by the ssDNA fragment GTGGCGGGGTGGCG in presence of the unlabeled TEN domain.

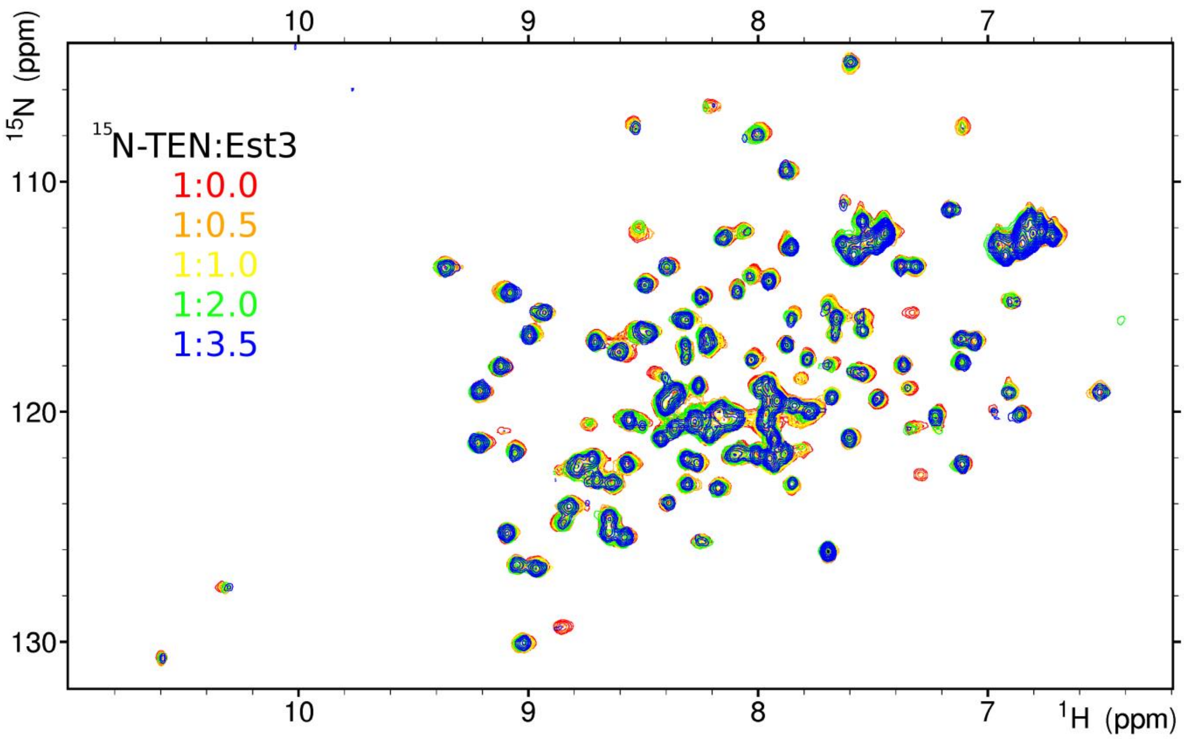

Figure S8. Titration of the ^{15}N-labelled TEN domain by the unlabeled HpEst3.

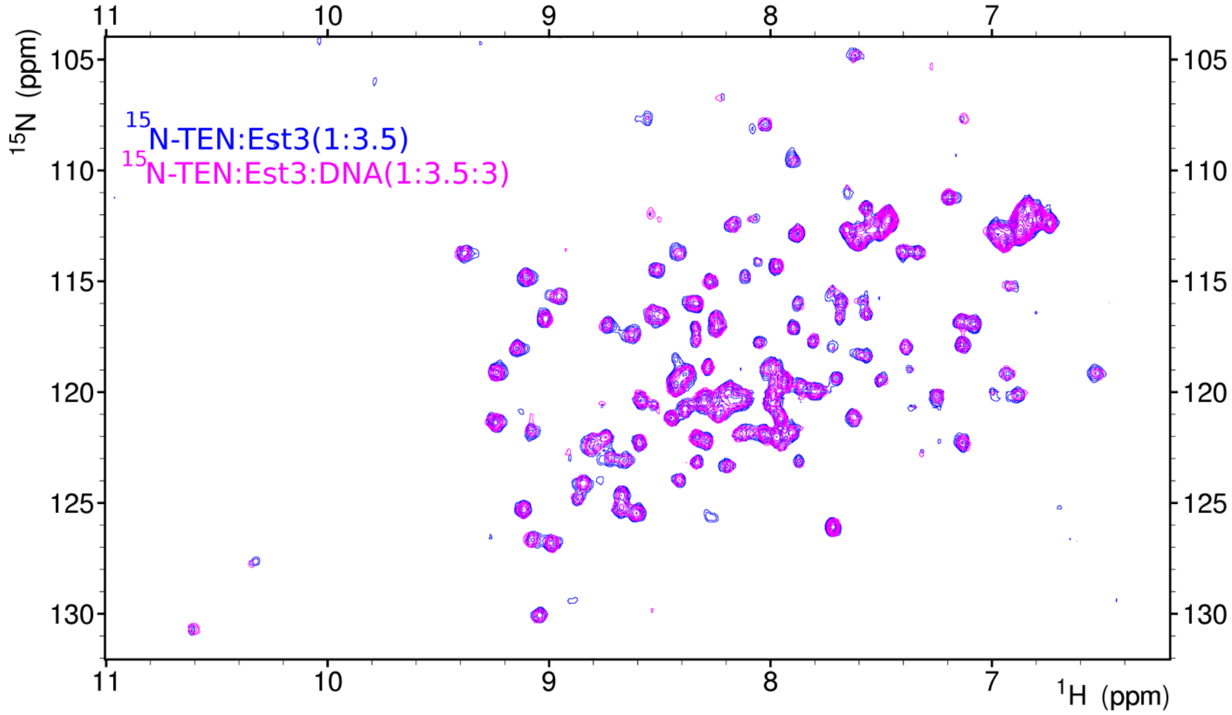

Figure S9. Titration of the ^{15}N-labelled TEN domain by the ssDNA GTGGCGGGGTGGCG in presence of the unlabeled HpEst3.

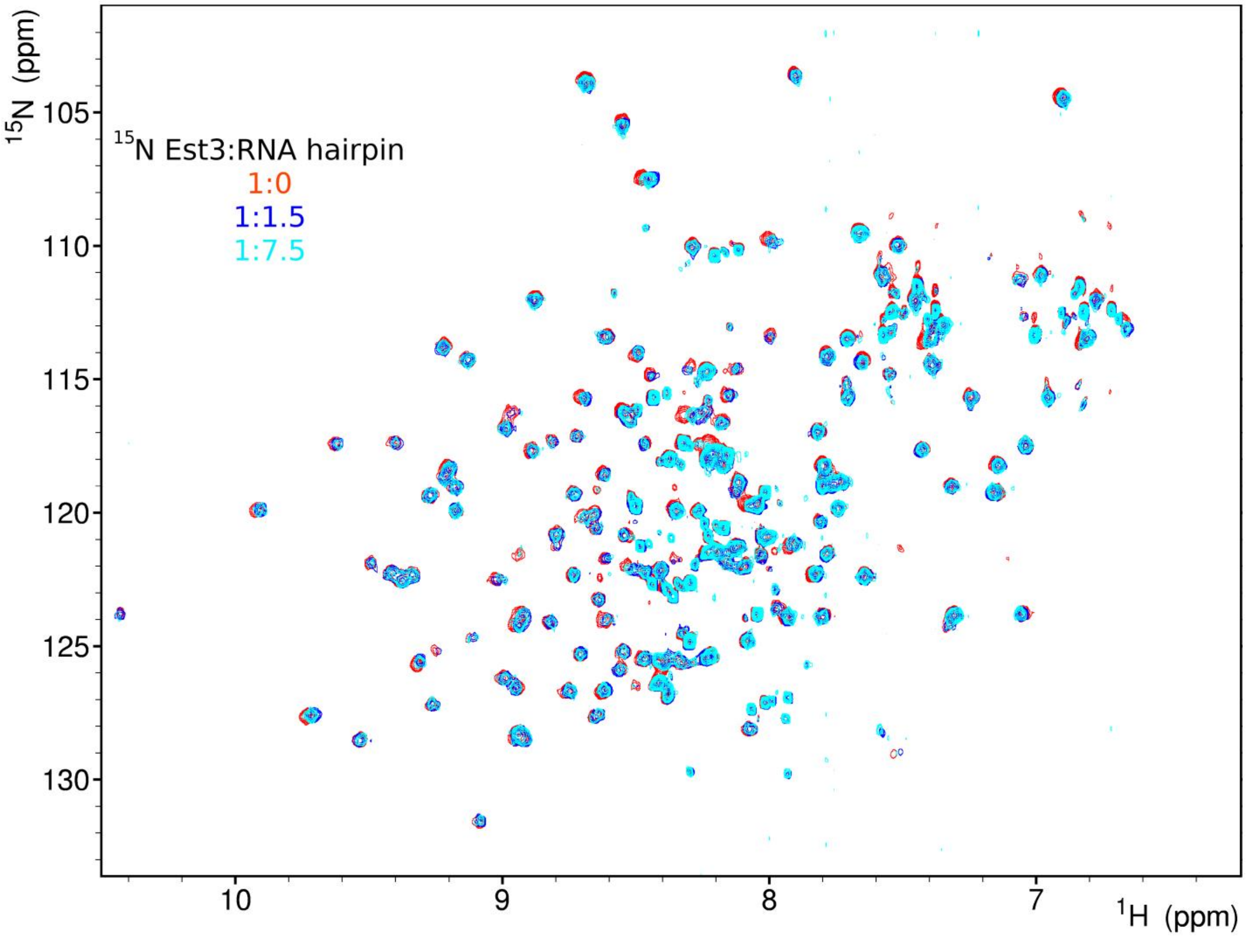

Figure S10. Titration of the ^{15}N-labelled HpEst3 by the RNA hairpin.

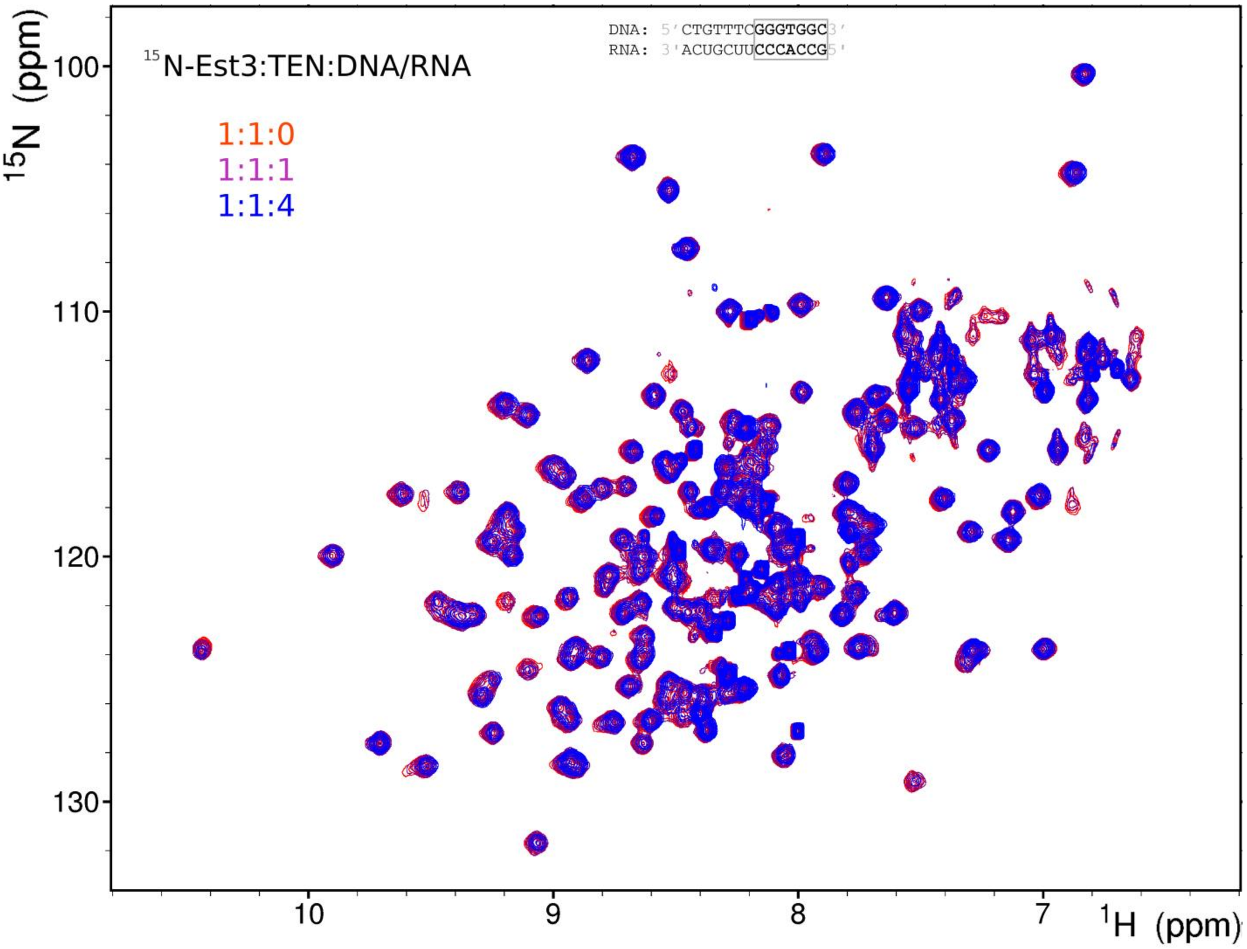

Figure S11. Titration of the ^{15}N-labelled TEN domain by the RNA-DNA fork.

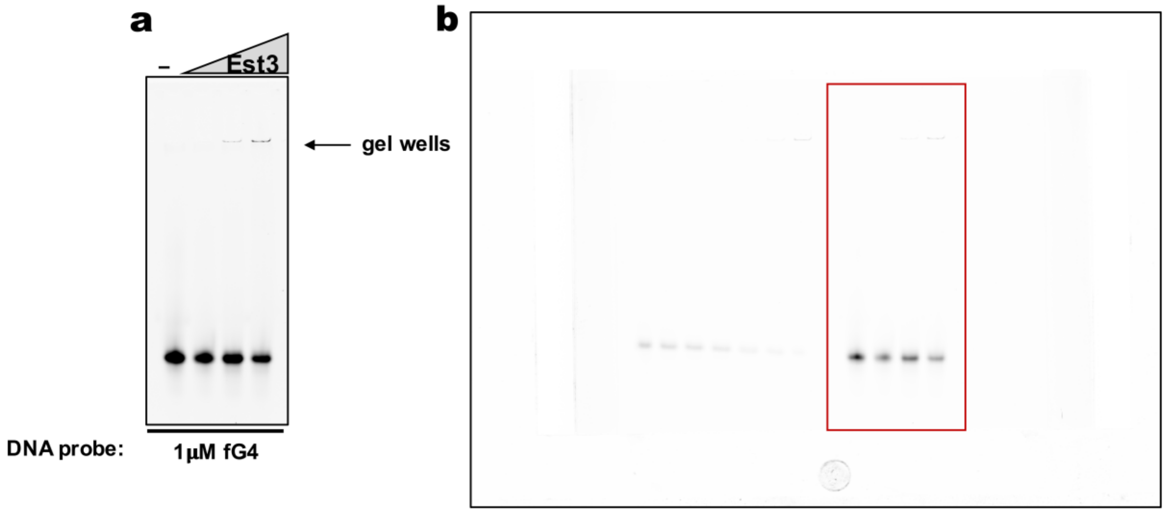

Figure S12. EMSA experiment with Est3. (a) 1 µM fG4 oligo was incubated with an increasing amount of Est3 (concentration range: 0, 1, 3, 10 µM). EMSA (b) The original gel from (a); red square shows the area, which is displayed in (a).

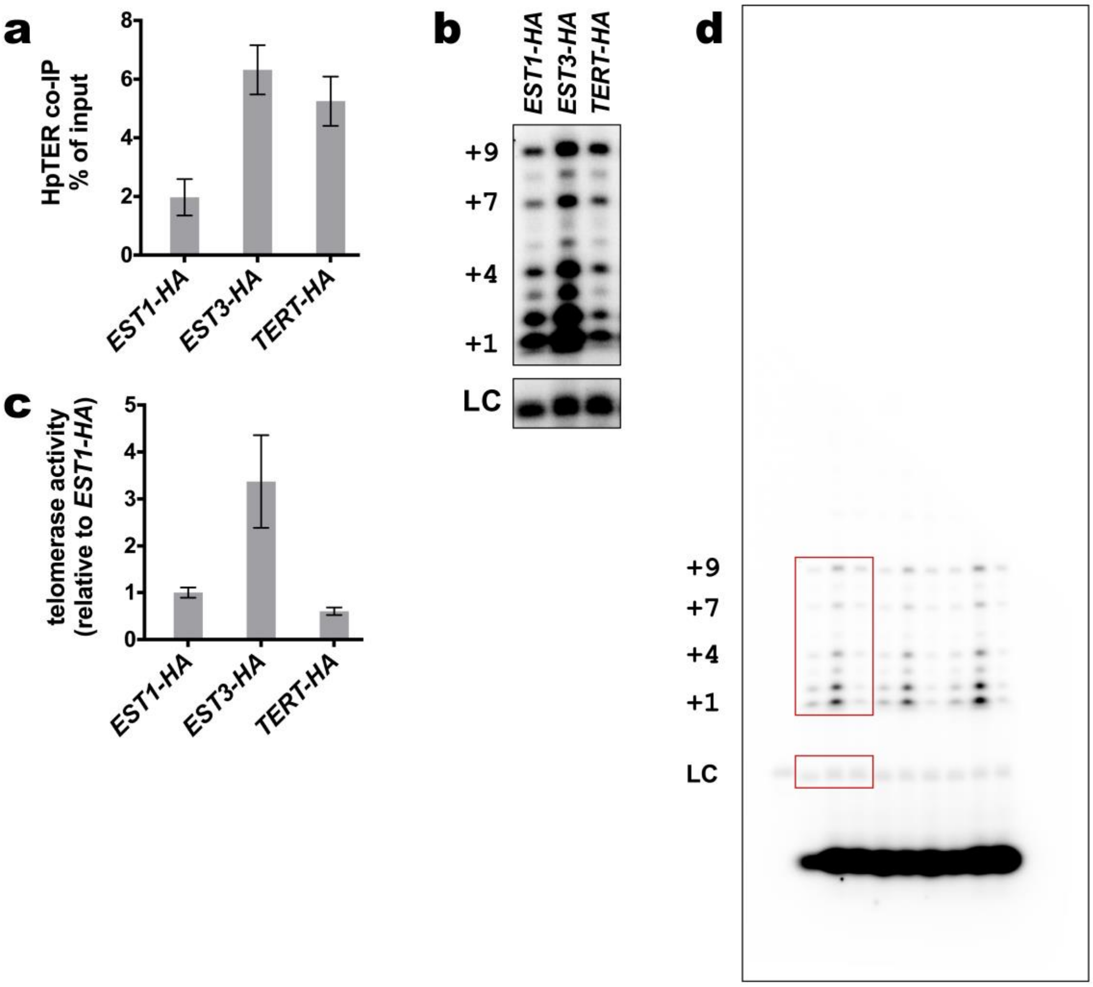

Figure S13. (a) Quantitative RT-PCR analysis of the HpTER co-precipitated on anti-HA agarose after incubation with extracts from the indicated strains. Three replicate samples of each strain were used for the experiment. Data represented as percentage of input RNA (mean ± SD). (b) Telomerase activity analysis of samples from (a). (c) Quantification of telomerase activity of samples from (b); the sum of the band intensities corresponding to telomerase products were normalized to the band intensities of the loading controls, value for *EST1-HA* sample was set to 1. We would like to note, that the apparent difference between telomerase activity in the samples may also stem from the fact that telomerase is immobilized on the affinity resin during primer extension. *TERT-HA* telomerase is more likely to be affected by such immobilization (compared to *EST1-HA* and *EST3-HA*), since it is directly involved in the catalysis, while direct role of Est1 and Est3 in the nucleotide addition is debatable (at least in *H. polymorpha*). (d) Original (full lane view; no contrast adjustment) gel for (b). Red squares show the areas, which are displayed in (b).

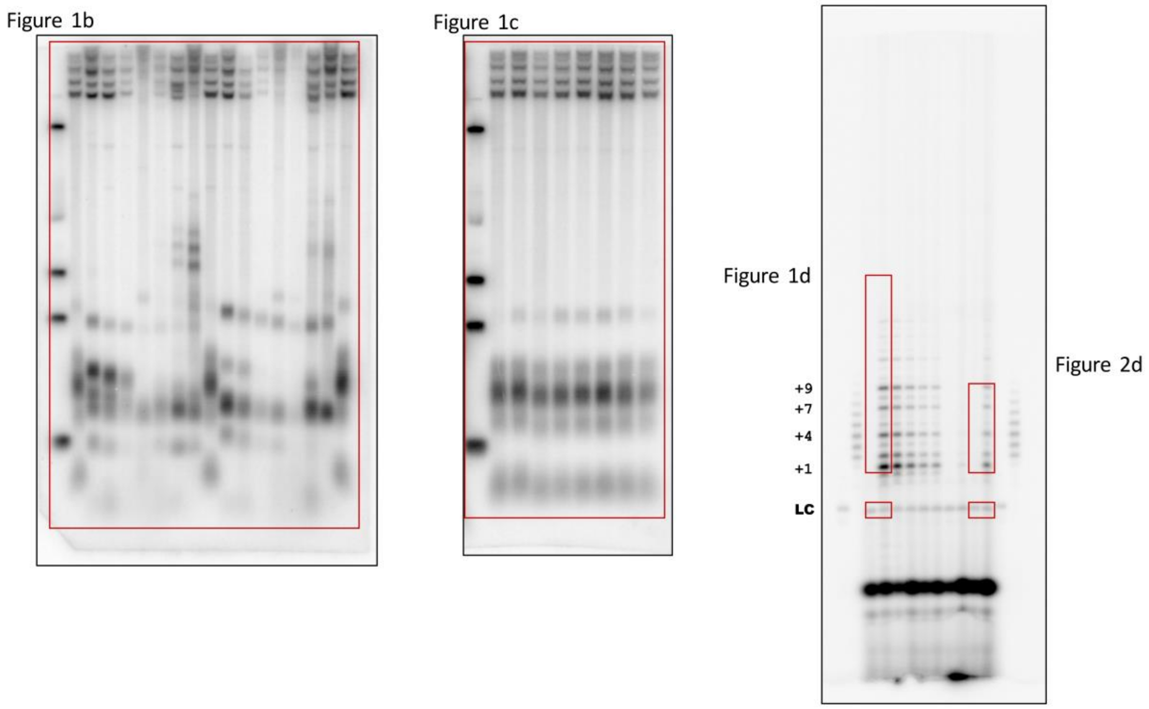

Figure S14. Original (full lane view; no contrast adjustment) gels/blots for Figure 1 and Figure 2d. Red squares show the areas, which are displayed in Figure 1 and Figure 2d. Please note, that the cropped portion of the gel shown in Figure 2d is flipped horizontally compared to the original gel presented here.

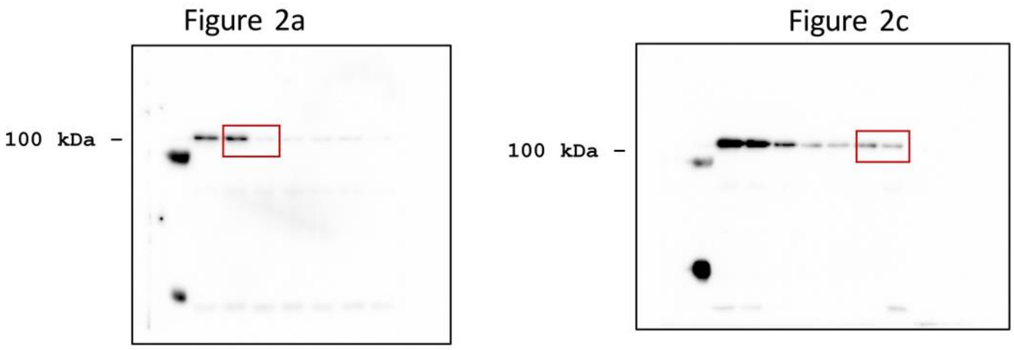

Figure S15. Original (full lane view; no contrast adjustment) gel/blots for Figure 2. Red squares show the areas, which are displayed in Figure 2.

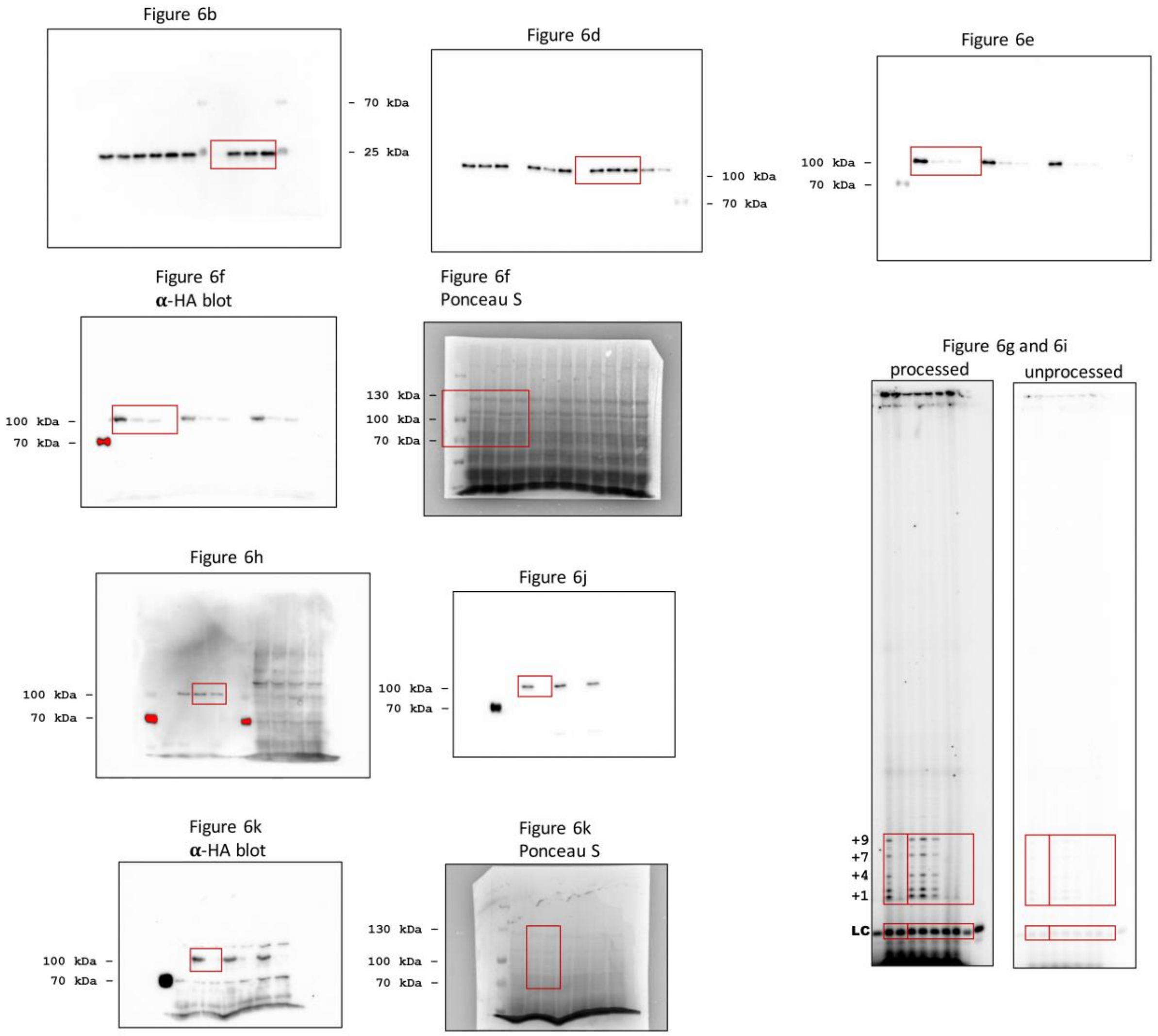

Figure S16. Original (full lane view; no contrast adjustment) gel/blots for Figure 6. Red squares show the areas, which are displayed in Figure 6.
